# Impact of active case-finding for tuberculosis on case-notifications in Blantyre, Malawi: A community-based cluster-randomised trial (SCALE)

**DOI:** 10.1371/journal.pgph.0002683

**Published:** 2023-12-05

**Authors:** Helena R. A. Feasey, McEwen Khundi, Rebecca Nzawa Soko, Christian Bottomley, Lingstone Chiume, Helen E. D. Burchett, Marriott Nliwasa, Hussein H. Twabi, James A. Mpunga, Peter MacPherson, Elizabeth L. Corbett

**Affiliations:** 1 London School of Hygiene and Tropical Medicine, London, United Kingdom; 2 African Institute for Development Policy, Lilongwe, Malawi; 3 Malawi-Liverpool-Wellcome Trust Clinical Research Programme, Blantyre, Malawi; 4 Helse Nord Tuberculosis Initiative, Kamuzu University of Health Sciences, Blantyre, Malawi; 5 National Tuberculosis Control Programme, Lilongwe, Malawi; 6 School of Health & Wellbeing, University of Glasgow, Glasgow, United Kingdom; University of Ottawa, CANADA

## Abstract

Active case-finding (ACF) for tuberculosis can help find the “missing millions” with undiagnosed tuberculosis. In a cluster-randomised trial, we investigated impact of ACF on case-notifications in Blantyre, Malawi, where ACF has been intensively implemented following 2014 estimates of ~1,000 per 100,000 adults with undiagnosed TB. Following a pre-intervention prevalence survey (May 2019 to March 2020), constrained randomisation allocated neighbourhoods to either door-to-door ACF (sputum microscopy for reported cough >2 weeks) or standard-of-care (SOC). Implementation was interrupted by COVID-19. Cluster-level bacteriologically-confirmed case-notification rate (CNR) ratio within 91 days of ACF was our redefined primary outcome; comparison between arms used Poisson regression with random effects. Secondary outcomes were 91-day CNR ratios comparing all tuberculosis registrations and all non-ACF registrations. Interrupted time series (ITS) analysis of CNRs in the SOC arm examined prevalence survey impact. (ISRCTN11400592). 72 clusters served by 10 study-supported tuberculosis registration centres were randomised to ACF (261,244 adults, 58,944 person-years follow-up) or SOC (256,713 adults, 52,805 person-years). Of 1,192 ACF participants, 13 (1.09%) were smear-positive. Within 91 days, 113 (42 bacteriologically-confirmed) and 108 (33 bacteriologically-confirmed) tuberculosis patients were identified as ACF or SOC cluster residents, respectively. There was no difference by arm, with adjusted 91-day CNR ratios 1.12 (95% CI: 0.61–2.07) for bacteriologically-confirmed tuberculosis; 0.93 (95% CI: 0.68–1.28) for all tuberculosis registrations; and 0.86 (95%CI: 0.63–1.16) for non-ACF (routinely) diagnosed. Of 7,905 ACF and 7,992 SOC pre-intervention survey participants, 12 (0.15%) and 17 (0.21%), respectively, had culture/Xpert-confirmed tuberculosis. ITS analysis showed no survey impact on SOC CNRs. Despite residual undiagnosed tuberculosis of 150 per 100,000 population, there was no increase in tuberculosis notifications from this previously successful approach targeting symptomatic disease, likely due to previous TB ACF and rapid declines in TB burden. In such settings, future ACF should focus on targeted outreach and demand creation, alongside optimised facility-based screening.

**Trial Registration**: ISRCTN11400592.

## Introduction

Tuberculosis remains a major killer, with 1.6 million deaths from TB in 2021, second only to COVID-19 as an infectious cause of death [1]. People living with HIV (PLHIV) have greatly increased susceptibility to active TB disease and death from TB [2], especially if their HIV is untreated, reflected in much higher *per capita* TB incidence and mortality rates in sub-Saharan Africa than other global regions since the 1990s. During the last decade, concerted investment to diagnose and treat PLHIV [3, 4] and reduce barriers to TB diagnosis has led to substantial TB epidemiology improvements [1, 5], although with setbacks due to service disruptions during COVID-19 [1, 6]. Regional TB incidence declined in Africa by an estimated 21% during 2015–21, although an estimated 4.1million people (980,000 in the WHO Africa region) with incident TB went undiagnosed and untreated in 2021 [1], 40% more than in 2019 [1]. Closing this treatment gap is essential to meeting TB elimination targets defined in the WHO EndTB Strategy targets [7] and may require more intensive systematic screening in facilities and also active case-finding (ACF) providing community-level diagnosis with focus on men and HIV-negative TB patients who otherwise tend to have prolonged duration of infectiousness [8].

ACF has potential to increase TB diagnosis and rapidly reduce the prevalence of undiagnosed infectious TB [9, 10] and was widely implemented in the last century [11]. ACF approaches vary greatly in intensity and delivery aspects, but often use periodic outreach by mobile teams using combinations of symptom screening, chest X-ray and either microscopy or, more recently, rapid molecular tests [10]. Less intensive, enhanced case-finding (ECF) uses health information or awareness campaigns to encourage health-seeking behaviour when people experience TB symptoms, with or without access to diagnostics at community-level [10]. Evidence of an indirect effect, for example health promotion, may be reflected in prolonged increased TB notifications due to a change in testing behaviour through increased knowledge of symptoms and diagnosis, reducing TB stigma, changing social norms, or providing a prompt for symptomatic people to attend a health facility for testing [12]. Because of the high cost, and risk of false-positive and false-negative screening results, community-wide ACF is conditionally recommended only for general populations with undiagnosed TB of 500 per 100,000 population or higher [10, 13]. Evaluating impact is technically difficult and costly, and a recent systematic review [9] identified just eight randomised controlled trials, only two of which had community TB infection incidence or prevalence outcomes [14, 15].

Sustainable Community Active-case finding for Lung Health (SCALE) was a cluster-randomised trial investigating the impact of ACF on cluster-level TB case-notification rates in Blantyre city, Malawi, with 2013–14 estimates of 1,014 per 100,000 adults with undiagnosed infectious TB [16]. We also investigated facility-based TB case-notifications following ACF in each cluster given potential to indirectly affect subsequent TB testing rates by health promotion [12].

## Methods

We conducted a parallel cluster-randomised trial (ISRCTN11400592) of TB ACF in high-density and peri-urban residential areas of Blantyre, Malawi with a pre-intervention prevalence survey implemented June 2019 to March 2020 [17]. 315 government community health worker catchment areas were aggregated to form the 72 trial clusters with an estimated 2015 population of ~5,200 adult (aged 15 years or older) residents in each cluster (~7,200 in 2019) and an overall estimated adult population of 372,000 (515,000 in 2019).

The trial was planned as a three-part study with pre- and post-intervention prevalence surveys to assess the effectiveness of three rounds of door-to-door community ACF delivered using brief door-to-door enquiry for prolonged cough with collection of two sputum specimens for microscopy [18, 19]. Blantyre city had an estimated prevalence of 1,014 per 100,000 adults with undiagnosed infectious TB in the 2013–14 Malawi national TB prevalence survey [20]. We had anticipated decline to 500 per 100,000 adults with undiagnosed TB due to previous ACF in Blantyre using the same approach by our team in 2011–14 [19] and national TB programme ACF 2015–2019, associated with declining TB notifications in Blantyre [5, 19].

The SCALE pre-intervention prevalence survey, however, showed a much greater than anticipated decline to 150–189 per 100,000 adults with undiagnosed TB [17], necessitating a change in primary outcome to cluster-level TB case-notification rates. The trial was then suspended following the onset of the COVID-19 pandemic after the pre-intervention prevalence survey and one round of ACF, with the primary outcome changed from prevalence of undiagnosed TB to evaluating the impact on bacteriologically-confirmed TB case notifications as described below.

### Study population

We conducted a city-wide census household enumeration with the Blantyre District Health Office (DHO) in 2015. In 2008 and 2018 the Malawi National Statistical Office (NSO) additionally conducted Population and Household National Censuses [21]. Adult (15 years and older) population denominators for this trial were estimated by applying estimated weekly population growth rates for each neighbourhood using linear interpolation and projection from the 2015 and 2018 data.

Prevalence survey participants were identified through random selection of 115 households, from a sampling frame of all household GPS co-ordinates obtained from Google Earth, aiming to recruit 215 adults (aged 18 and above) per cluster. All adults from selected households who were willing and able to provide consent were included in the prevalence survey. For the ACF intervention, all adult residents (18 years and older) living in intervention clusters, with a cough of two weeks or more and not currently on TB treatment were eligible.

### Procedures

A pre-intervention prevalence survey was conducted in all clusters (two clusters per week) and the ACF was conducted in the intervention arm the following week with staggered initiation over a period from 12 May 2019 until 2 March 2020. Local leaders were engaged and study information meetings held in all clusters prior to the start of the prevalence survey. For the prevalence survey, participants from randomly selected households were invited to attend a study tent located in an accessible neighbourhood location for digital chest X-ray, interpreted by a trained radiographer (any abnormality versus normal), with assistance by computer aided diagnostic software (Qure.ai version 2). Participants reporting a cough of any duration or an abnormal X-ray were asked to provide two spot sputum samples for smear microscopy, Xpert MTB/RIF and Mycobacteria growth indicator tube (MGIT) culture. Confirmatory samples were requested from participants with positive TB test results, with support to register for TB treatment at their nearest health facility. HIV testing was offered to all participants using OraQuick (OraSure) and Determine (Alere) finger-prick rapid HIV diagnostic tests conducted in parallel, with Uni-Gold (Trinity Biotech) to confirm positive HIV results. Prevalence survey activities took five to six days per cluster.

### Active-case finding

The ACF intervention commenced in intervention clusters two- to three-days after the prevalence survey, and lasted for a period of up to five days. Fieldworkers moved door-to-door leaving information leaflets and enquiring about symptoms of cough lasting two weeks or longer in any adult household member, including those not present at the time of ACF team visit. GPS coordinates were taken to document each household visit. Adults with cough were asked to provide a spot sputum and given a sputum pot for next-morning sputum collection. Information leaflets and two sputum pots were provided for reported symptomatic–but absent–household members, with a leaflet explaining how to collect sputum. Sputum samples were collected by the ACF team the next-day and examined using smear microscopy. Up to three visits were made per household if no one was present, with information leaflets left at all households. Participants with positive microscopy results were contacted directly by telephone and household visit, asked for a confirmatory sample, and assisted to register for TB treatment at the nearest health facility. For participants with negative results, a neighbourhood tent was set up on a designated day in the following calendar week to issue results.

### Standard of care

All public medical services are provided free at the point of care in Malawi. To provide an enhanced standard of care to all residents, study clinic assistants were assigned to each of 10 District Health Office (DHO) Blantyre primary health facilities between May 2019 to October 2020. These Clinic Assistants assisted the District Health TB officers in their duties and facilitated identification of outpatient clinic attendees with cough through triage and referral to clinical officers if eligible for TB investigations under National TB Programme (NTP) guidelines.

### Outcomes

The primary outcome of the final protocol was the cluster-level case notification rate (CNR) of bacteriologically-confirmed TB (per 1000 adult residents) in the 91 days after the start of the prevalence survey. Secondary outcomes were the cluster-level CNR for all-form TB and CNRs for both bacteriologically-confirmed and all-form TB identified through routine diagnosis (excluding those identified through the ACF and prevalence survey) during the same period.

Case notifications were recorded through a tablet-based electronic TB database, established in 2011, delivered by DHO TB officers, and maintained jointly by Blantyre DHO and Malawi-Liverpool Wellcome Programme (MLW), which recorded details for all patients registering for TB treatment within urban Blantyre [5, 22], including place of residence GPS co-ordinates identified through a satellite map application (ePAL), which has previously been validated [22, 23]). All patients registering for TB treatment were asked for a spot sputum sample for smear microscopy and MGIT culture at the MLW/Kamuzu University of Health Sciences (KUHeS) TB Research Laboratory. The electronic registry was reconciled with NTP paper registers on a monthly basis and household GPS co-ordinates of 5% of participants were checked through home visits. This routine case notification data records those aged 15 years and above as adults, whereas the intervention was only offered to those aged 18+.

Data was censored from 23 March 2020 (date of declaration of Malawi COVID-19 state emergency) since COVID-19 led to a large reduction in TB case-notifications in Blantyre [5] and elsewhere. This was 21 days after the start of the prevalence survey in the final two clusters. To anonymise data all GPS co-ordinates were removed once cluster and trial arm were identified.

### Randomisation and masking

Clusters were randomly assigned (1:1) to receive either the door-to-door ACF intervention, or enhanced standard of care. Randomisation was conducted at a public meeting using random selection of one number from a previously prepared list of 999 randomly selected allocations generated by the trial statistician using a computer programme. Randomisation was constrained to provide balance on mean distance from cluster centres to the nearest health clinic, baseline TB case-notification rates, adult population, longitude and latitude of cluster centres, and referral health centre. Participants and field-workers were not masked to the intervention, but laboratory work and clinical management were completed without reference to trial arm and analysis by trial arm was not undertaken until the final analysis.

### Statistical methods

Sample sizes were initially calculated to provide ≥80% power to detect a 28 to 35% reduction in the original trial outcome of prevalence of undiagnosed TB. With the revised primary outcome the study was expected to provide 89% power to detect a 30% increase in bacteriologically-confirmed case-notifications (based on previous interventions in Blantyre [19]), assuming a rate of 276 cases per 100,000 adults in the control arm, 91 days (13 weeks) of follow-up per cluster and an intra-cluster correlation of 0.3.

For the primary outcome (bacteriologically-confirmed TB CNRs), we calculated the cluster-specific number of people with bacteriologically-confirmed TB identified as initiating TB treatment by the ePAL system in the 91 days after the start of the prevalence survey in that cluster, and divided by the estimated person-years of follow-up in each cluster to give CNRs per 100,000 person-years. The CNR ratio, adjusted to account for the variables that randomisation was constrained by, was estimated through a Poisson regression model with random effects to account for clustering. Secondary outcomes were calculated similarly using the relevant numerators obtained from ePAL treatment registrations.

Time trends in CNRs were plotted, calculating the five-week rolling mean case notification rates, with 95% confidence intervals estimated through 1,000 bootstrap replications, by arm and stratified by sex. In a further pre-planned analysis of impact of the prevalence survey alone on case notification rate over time, an interrupted time series analysis was conducted on the case notification rates over the 52 weeks before and 13 weeks (91 days) after the start of the prevalence survey. The Poisson regression model included a linear term to account for time trend and two indicators to model the impact of the prevalence survey. One indicator was used to estimate impact in the 6-week period immediately following the survey and the other was used to estimate long-term impact. Newey West confidence intervals were calculated to account for over dispersion and auto-correlation [24].

All analyses were done with R version 4.2.1. This trial is registered with the ISRCTN registry (ISRCTN11400592).

### Data and reproducibility

Data and code to reproduce this analysis is available from https://osf.io/fvqtw/.

### Ethics

Approval was granted by the research ethics committees of the Malawi College of Medicine (now Kamuzu University of Health Sciences) and the London School of Hygiene and Tropical Medicine. Written (or witnessed if illiterate) informed consent was provided by all participants in the prevalence survey and active case-finding. Oral consent (or assent with oral consent from parent/guardian for those aged under 18) was provided by people registering for TB treatment for electronic data capture, including recording of household co-ordinates. Oral consent and assent were used for the latter since the electronic register data capture was conducted as part of normal clinical practice by District TB Officers. Approval was sought and gained from the District Health Office and local chiefs for the study to be conducted in their area, and locally nominated volunteers supported the delivery of the intervention in each ACF cluster.

## Results

### Participant and cluster characteristics

36 clusters were randomised to each study arm with an estimated 2019 adult population (age 15+) of 261,244 in the ACF clusters and 256,713 in SOC clusters. Clusters were followed up for a median of 168 days (range 21–316) and people in ACF and SOC clusters contributed 58,944 and 52,805 person-years of follow-up, respectively (Table 1).

**Table 1:** Population denominators and baseline cluster characteristics identified through pre-intervention prevalence survey by study arm*.

Unit	Variable	Unit / category	ACF	SOC
Community		clusters	36	36
	Completed 91 days FU**	clusters	29	26
	Adult population in 2015	(100)	1899	1821
	Previous CNR	(per 100 000 PYs)	262.0	275.9
	Adult population in 2019	(100)	2612	2567
	Adult person years ***	(PYs)	58944	52805
	Distance to health facility	Metres (mean, range)	889.8 (84.5–3022.5)	938.7 (106.7–2675.1)
Household				
	Crowding	persons per room (mean, range)	0.93 (0.10–4)	0.96 (0.14–4)
	SES indicators	bottom quartile (%)	24.3	25.7
Individual				
	Age	years (mean,range)	32.2 (18–94)	32.6 (18–98)
	Sex	Male (%)	47.0	46.4
	HIV/ART status	HIV+ on ART (%)	10.6	11.0
		HIV+ not on ART (%)	1.4	1.6
		HIV unknown (%)	4.3	3.9
	TB contact (within 12 months)	Yes (%)	5.0	4.0
	Previous TB treatment	Yes (%)	2.7	2.8
	Reported TB symptoms†	Cough (any duration) (%)	3.5	3.4
		Cough ≥ 2 weeks†† (%)	2.0	2.0
		Night sweats (%)	5.3	4.7
		Weight loss (%)	5.6	5.0
		Fever (%)	3.2	2.5
		Any (cough any duration) (%)	13.7	12.8

* % for categorical data; mean (range) for quantitative data

** Number of clusters completed 91 days follow-up after prevalence survey before 23 March 2020

*** Person years of follow-up after prevalence survey

^†^ Data based on complete case analysis (completed records) for preceding prevalence survey participants (imputed alterative estimates adjusted for incomplete participation are described more fully in [17])

^††^ Chronic cough

Note: Original data for categorical variables presented in Table A in S1 Text

Of the 36 clusters in each arm, 29 of those in the ACF arm and 26 in the SOC arm completed 91 days after the start of the prevalence survey before 23 March 2020, when data was censored (Table 1). Data from all clusters was included in the analysis, independent of follow-up time. Baseline characteristics of the adult population are presented by arm in Table 1. Household characteristics and most individual characteristics (sex, age, HIV status, previous TB treatment and reported TB symptoms) were similar between arms.

### Pre-intervention prevalence survey

Between 12 May 2019 and 13 March 2020, 15,897 participants were recruited to the pre-intervention prevalence survey (7,905 in the ACF arm and 7,992 in SOC clusters). Overall, 1,274/15,897 (8.0%) TB presumptive participants (cough and/or abnormal X-ray) and 29 (0.18%) bacteriologically-confirmed cases of TB were identified: 12 (151 per 100,000, 95% CI: 87–265) in the ACF arm and 17 (213 per 100,000, 95% CI: 133–340) in SOC clusters. All were supported to register for TB treatment locally except four in the SOC arm who moved and registered in treatment sites outside of Blantyre.

Clinical and microbiological details of the 29 bacteriologically-confirmed cases are presented in Table B in S1 Text.

### ACF intervention

**Fig 1:**
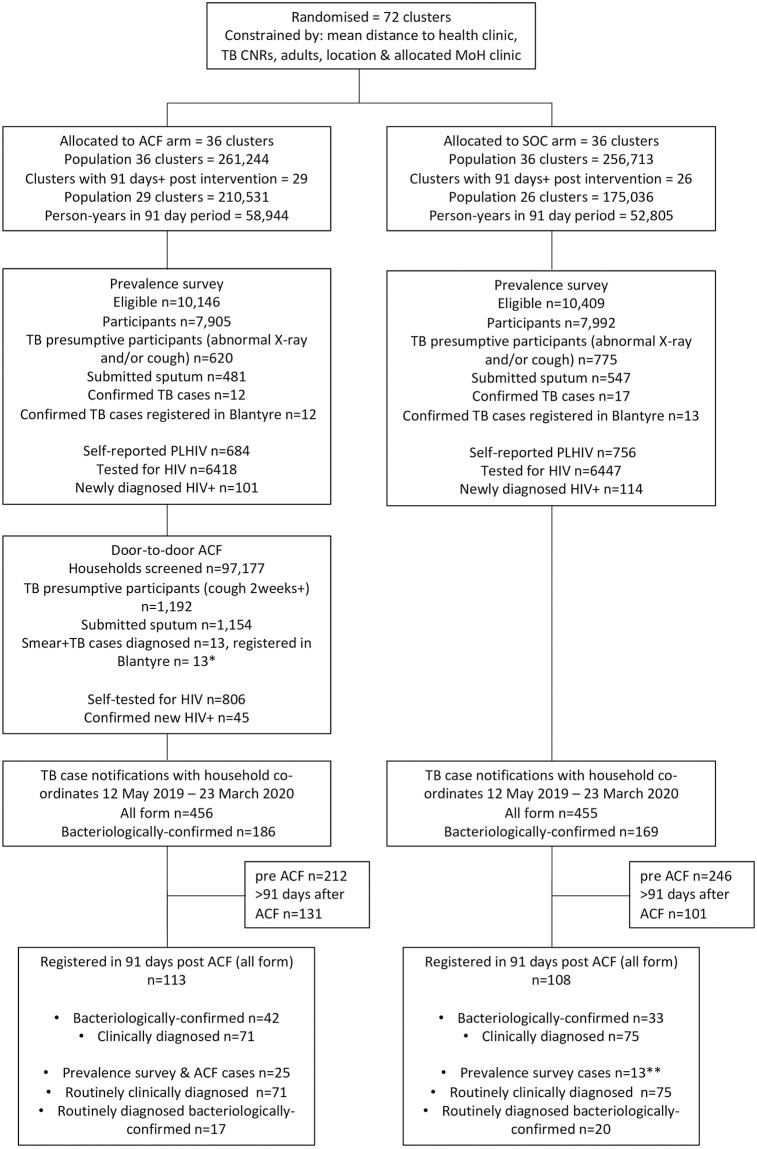
Consort diagram of trial participants. * Of 13 ACF-identified smear+ TB cases 10 were HIV positive when registering for TB treatment.

Between 19 May 2019 and 14 March 2020 the door-to-door ACF intervention visited 97,177 households with 261,244 adult (15 years or older) residents. In total, 1,192 (0.5% (1,192/261,244) adults volunteered or were identified by household members as having chronic cough–substantially below the 2.0% of adults who reported cough of 2 weeks or longer on direct enquiry for symptoms in the pre-intervention prevalence survey (Table 1). Of those identified by household members, 1,154 (96.8%) submitted sputum; 13/1,192 (1.1%) were smear positive (Fig 1). All participants were confirmed positive by Xpert and MGIT culture (Table C in S1 Text). The yield of the ACF intervention was therefore 5.0 new confirmed TB cases per 100,000 adult population.

During the study period, 1,475 adults aged 15 years or older registered for TB treatment through any diagnostic route at health facilities in urban Blantyre. Of these 911/1,475 (61.8%) were resident within SCALE clusters and had their household GPS co-ordinates recorded. 368/1,475 (25.0%) urban residents had no co-ordinates recorded (reason unknown) and of those with co-ordinates recorded, 196/1,475 (13.3%) were resident outside of SCALE clusters (i.e. lived in urban Blantyre, but not in a study cluster). Of these registering cases, 456/911 (50.1%) were resident in the ACF arm, (186 [40.8%] bacteriologically-confirmed), and 455/911 (49.9%) were resident in the SOC arm (169 [37.14%] bacteriologically confirmed)–Fig 1. In ACF clusters, 113 cases were registered in the 91 days after the start of the intervention (42 [37.2%] bacteriologically-confirmed). For SOC clusters, 108 cases (with 33 [30.6%] bacteriologically-confirmed) were registered in the same 91-day period.

Overall, across arms, of all cluster-residents registering for treatment for bacteriologically-confirmed TB through any diagnostic route, 69.9% (248/355) were male, 51.0% (181/355) were HIV positive (94.5% were taking ART), and mean age was 35 years (SD 12)

### Outcomes

The case notification rate (CNR) for bacteriologically-confirmed TB registered through any diagnostic route in the intervention arm during the 91 days after the start of the intervention was 71.3 per 100,000 adults per year (42/58,944) and 62.5 per 100,000 adults per year in the SOC arm (33/52,805), giving an unadjusted rate ratio of 1.14 (95% CI: 0.72–1.80, p = 0.58)–Table 2. The adjusted rate ratio was 1.12 (95% CI: 0.61–2.07, p = 0.71).

**Table 2:** Primary and secondary outcomes: TB case-notification rates (any diagnostic route) at 91 days*.

Endpoint			Unadjusted			Adjusted**		
	ACF	SOC	Ratio+	95% CI	P-value	Ratio	95% CI	P-value
*Adult CNRs (91 days)*								
Bact-confirmed	42/58944†	33/52805	1.14	0.72–1.80	0.58	1.12	0.61–2.07	0.71
All TB registrations	113/58944	108/52805	0.94	0.72–1.22	0.63	0.93	0.68–1.28	0.67
All routinely diagnosed TB††	88/58944	95/52805	0.83	0.62–1.11	0.21	0.86	0.63–1.16	0.33
*Other pre-set CNRs*								
Bact-confirmed routinely diagnosed	17/58944	20/52805	0.76	0.40–1.45	0.41	0.73	0.36–1.47	0.37

* CNR based on routine notification data using enhanced surveillance system (ePAL) for GPS data

^+^ Ratios obtained by fitting a negative binomial regression model to cluster-levels counts with the number of person years included as an offset.

** Adjusted for variables used to restrict randomisation: Previous CNR, number of adults, mean distance from cluster centres to the nearest health clinic, allocated health centre and longitude and latitude of cluster centre

^†^ Number of notifications within 91 days / person-years follow-up

^††^ Routinely diagnosed TB excludes ACF and prevalence survey participants, aiming to measure “indirect effect” of ACF

For the secondary outcome of all forms of TB, CNRs were 191.7 per 100,000 adults per year in the ACF arm, and 204.5 per 100,000 adults in the SOC arm, with an adjusted rate ratio of 0.93 (95% CI: 0.68–1.28, p = 0.67)–Table 2. For routinely diagnosed all forms of TB (excluding those detected by the prevalence survey and ACF interventions), CNRs for the ACF arm and SOC arms were 149.3 per 100,000 adults per year and 179.9 per 100,000 adults per year, respectively (adjusted rate ratio: 0.86, 95%CI: 0.63–1.16, p = 0.33). Comparison of bacteriologically-confirmed routinely diagnosed case notifications gave an adjusted CNR ratio of 0.73 (0.36–1.47, p = 0.37).

### Time trend analysis

**Fig 2:**
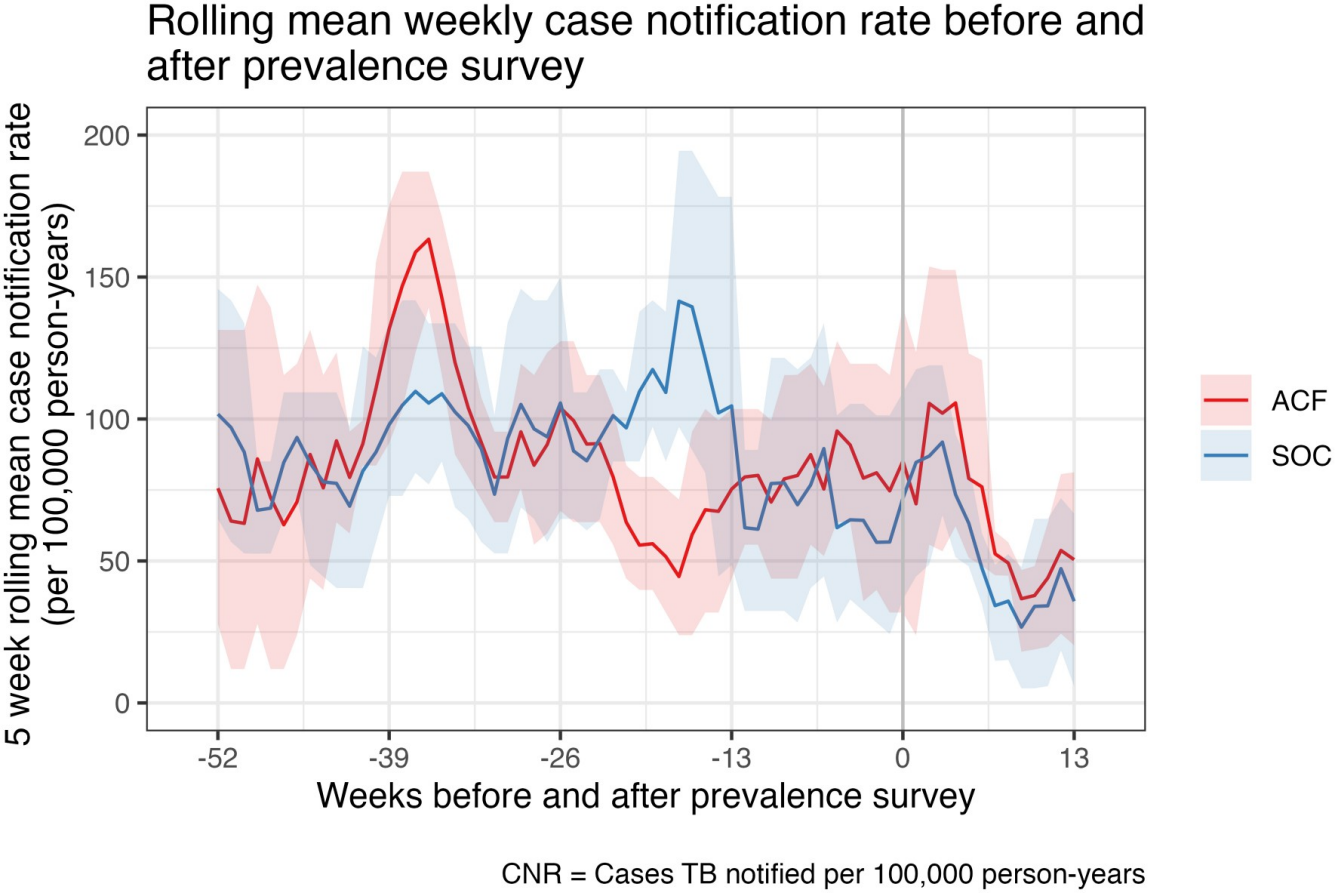
Rolling mean weekly case notification rate before and after prevalence survey.

**Fig 3:**
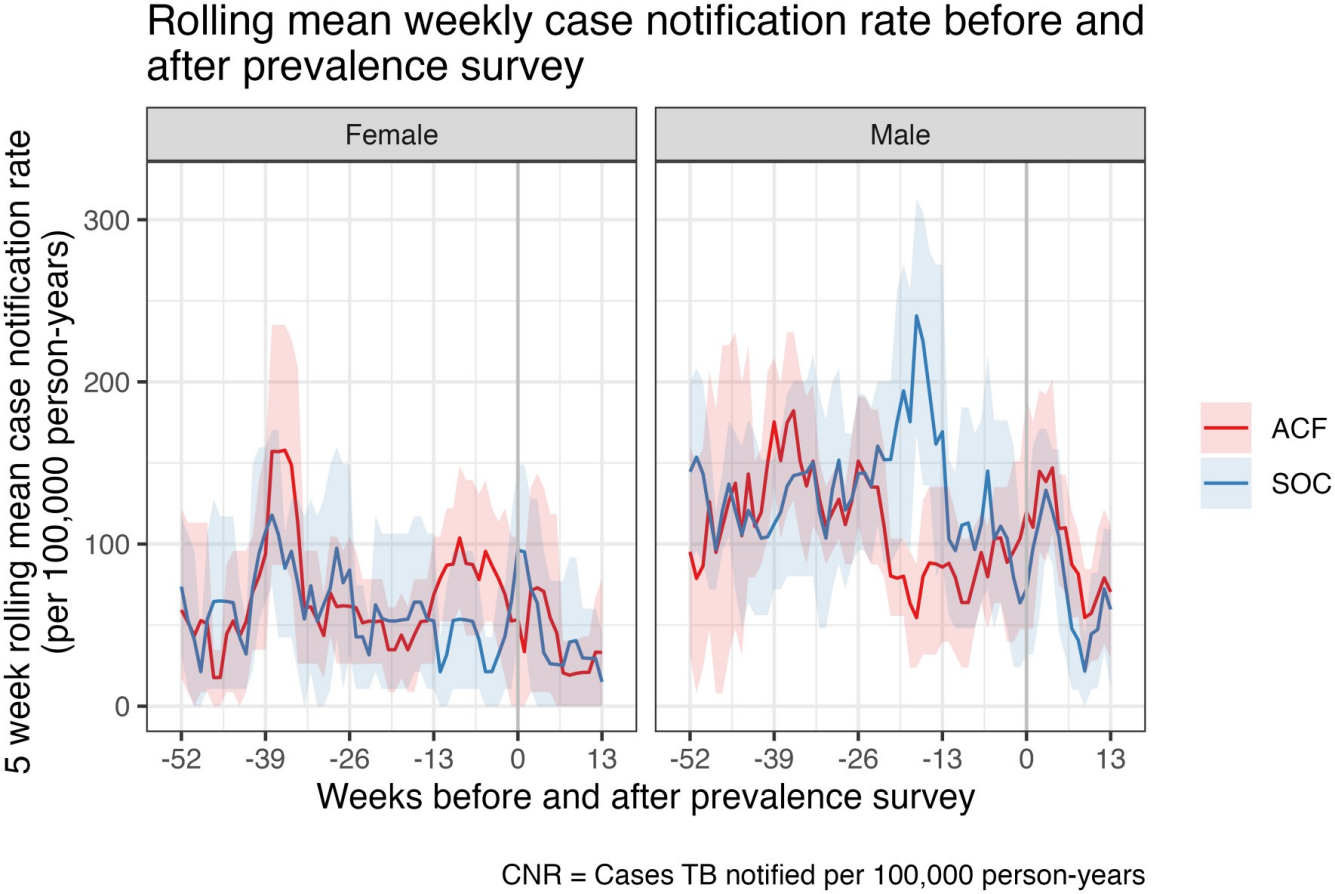
Rolling mean weekly case notification rate before and after prevalence survey by sex.

In the period from 52 weeks before to 13 weeks after the intervention, the five-week rolling mean estimated annual bacteriologically-confirmed CNR varied from 36.6 (95% CI: 18.2–46.7) to 162.6 (95% CI: 135.4–187.1) per 100,000 adult years in the ACF arm, and from 26.8 (95% CI: 5.2–46.5) to 141.3 (95% CI: 97.2–190.5) per 100,000 adult years in the SOC arm (Fig 2). Higher CNRs were observed among men (overall mean CNRs 105.9 [ACF arm] and 120.3 [SOC arm] per 100,000 person years) compared to women (overall mean CNRs 61.1 [ACF] and 55.6 [SOC] per 100,000 person years) during this period (Fig 3). Substantial week-to-week variation reflects small numbers of mean cases per arm per week. No overall time trends were observed for both bacteriologically-confirmed and all-forms of TB CNRs.

During the 52 weeks prior to the prevalence survey in the SOC clusters, 506 TB case notifications were recorded with GPS co-ordinates. In the 13 weeks after the prevalence survey started there were 109 notifications: 61 of these in the first 6 weeks and 48 in the subsequent 7 weeks. The mean weekly CNR pre-intervention was 186.5 (range 72.8–336.2) per 100,000 adult years before the intervention, 189.5 (range 108.4–287.0) per 100,000 adult years in the six weeks immediately after the survey started, and 163.5 (range 52.6–351.8) per 100,000 adult years in the subsequent 7 weeks.

**Fig 4:**
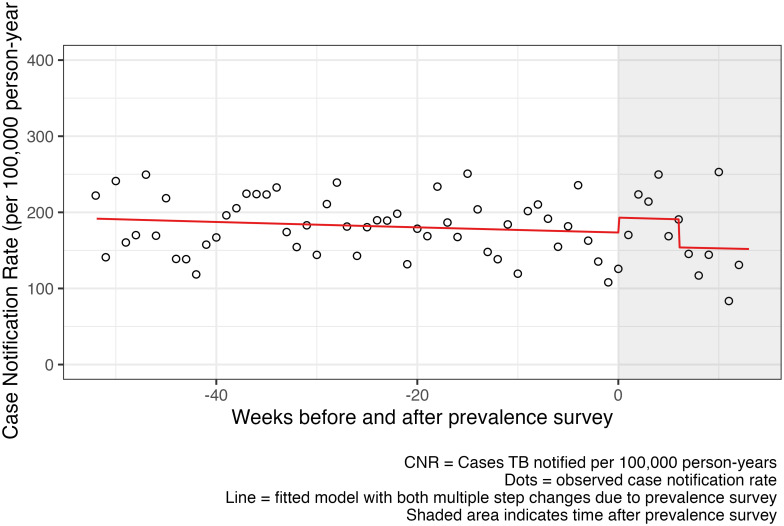
SOC arm case notification rate before and after prevalence survey.

The start of the prevalence survey was associated with a 4.9% (95% CI -20.5% to 38.5%, p-value = 0.8) increase in TB notifications from cluster residents, which then reduced to -9.2% (95% CI -33.5% to 23.8%), p-value = 0.6) lower than the pre-intervention level after 6 weeks, until the end of the analysis period (Fig 4).

## Discussion

In this cluster-randomised trial investigating the impact of ACF on case-notifications, we found no evidence of effectiveness of door-to-door enquiry for cough of 2 weeks or longer, an approach previously shown to increase case-notification rates when first implemented in the current trial setting of Blantyre, Malawi in 2011–14 [19] and in other African settings [18, 25]. The effectiveness of the intervention was likely limited by low participation and lower-than-expected prevalence of undiagnosed TB. TB surveillance needs to diversify to allow programmes to track, adapt and better target interventions towards the remaining people with undiagnosed TB in a more precise and timely fashion. To achieve this, ACF interventions should have robust impact assessment, such as the geolocated case-notification approach used here, and future ACF in Blantyre should be more highly targeted to defined sub-populations, such as working age men, as a complement to optimised health centre screening and laboratory strengthening.

Our ACF approach targeted symptomatic individuals as a less costly, but less sensitive, alternative to systematic screening regardless of symptoms, with targets based on population-level estimates of chronic cough. A critical limiting factor in SCALE, however, was low participation: only 23% of ACF cluster residents with chronic cough (estimated from prevalence survey immediately prior to the intervention) submitted sputum, lower than for the same strategy in the same city in 2011–14 [19], despite undiagnosed infectious TB remaining well above TB elimination targets affecting 150–189 per 100,000 adults [17]. Declining ACF participation has been noted previously during prolonged or repeated implementation [15, 18] and may indicate community fatigue as ACF becomes less novel, or as TB becomes a less pressing community concern as true TB incidence and mortality rates fall. In addition, since historical TB screening activity in Blantyre has often used a symptom screening approach similar to that used in this study, much of the TB responsive to this form of ACF (symptomatic or clinical) may have already been detected and the pool of undiagnosed people willing to participate in this type of intervention already depleted [26].

Undiagnosed infectious TB in Blantyre has been reduced from over 1,000 to 150–189 per 100,000 adults, associated with TB interventions focused on symptomatic disease, including ACF, decentralisation of TB diagnostic centres and more use of molecular diagnostics as well as scale up of HIV services. This decline is also reflected in decreasing case notifications and concurrent evidence of declining TB burden in primary care attendees [27, 28]. Blantyre is now below the threshold (500 per 100,000 adults) for which community-wide intervention is recommended [10], but remaining prevalence still suggests need for affordable and effective ways to focus case-detection, for example targeted spatially [29] or by target group [10], such as adult men, given their increased prevalence of undiagnosed TB [30]. The ACF approaches used over the last 20–30 years, such as the door-to-door strategy used here, now need to be adapted but research is needed to identify optimal intervention and evaluation approaches.

ACF intensity can be increased by more systematic screening, more sensitive diagnostic algorithms based on molecular sputum tests and digital chest X-ray with computer-aided diagnostics (DCXR-CAD) [10, 31], or higher intensity intervention [9, 32]. High intensity ACF, notably annual sputum molecular testing, is effective [15, 33] but costly, and should only be considered in medium-burden settings when more efficient alternatives such as facility-based systematic screening [34, 35] and diagnostic cascades have been optimised [36]. Alternatively, national TB programmes in settings such as Blantyre could start to explore community-led and peer-led approaches that have been successfully used to target and obtain high participation by high-risk populations for HIV testing, including men [37]. With potential for effective self-sampling approaches, such as tongue swabs [38], programmes may have to choose whether to maximise reach to previously untested high-risk individuals, at the cost of sensitivity, or using more highly sensitive universal approaches in communities [15, 33] despite the difficulties and cost of scale-up and maintenance [39].

To ensure we know which ACF approaches are the most effective we need to robustly measure the impact of TB screening interventions, supported by digital technologies such as the high-resolution surveillance of GPS locations of TB case notifications used in SCALE. To our knowledge this is only the second published trial of ACF, after a study by Miller et al in Brazilian favelas [40], to assess the impact on TB case notifications in the period after–instead of only during–intervention implementation. In other studies using impact of TB case-notifications as the outcome the time period of analysis is the overall calendar period for the ACF intervention implementation even though the interventions cover a large area and are usually implemented in a staggered fashion [9]. Whether randomised controlled trials, before-after studies (where the comparison is just over time) or controlled before-after (with a parallel control group) studies they examine the impact during rather than after the ACF. Our enhanced surveillance system, however, enabled us to identify the timing of each case notification relative to when the ACF was conducted in that residential cluster, providing the temporal component needed for casual inference. To strengthen evidence generated, future trials should have robust assessment methodologies and report outcomes relative to the dates of the ACF intervention in that specific area.

We found no significant impact of the ACF intervention or community TB prevalence survey (which is also a form of ACF, since it aimed to identify undiagnosed TB) on TB case-notifications, although our study had insufficient power to fully assess whether the observed 12% increase in bacteriologically-confirmed case notifications was significant or not. Our analysis of time trends suggests that there may have been a small peak in TB case notification rate after the intervention/prevalence survey in both arms followed by a dip, as would be expected with the substitution effect, whereby patients who would otherwise have been diagnosed routinely during the intervention period and immediately afterwards are instead found though ACF [12]. This substitution effect could account for the observed non-significant 12% increase in bacteriologically-confirmed case notifications and non-significant decreased case notifications for all registrations and all form TB.

Routine facility-based TB case notifications also showed no indication of any indirect effect (such as health promotion) during ACF. This result does not preclude an indirect impact though, as this could be masked by the substitution effect, but other outcome evaluations (TB testing, TB knowledge, attitudes and perceptions, qualitative research) are needed to identify if this occurs and future interventions should continue to monitor any indirect effects [10]. This lack of observed impact could also reflect previous ACF interventions having met the accumulated demand [41] and a subsequent lack of novelty for targeted populations.

Regardless of future ACF intervention choices, current TB surveillance is not providing the richness and timeliness of data needed to enable many national programmes to evaluate impact, change strategy as and when needed as local TB epidemics become increasingly concentrated [26]. Surveys of TB immunoreactivity were used for surveillance of TB epidemics [42], and could be reintroduced with newer tests [43]. National programmes in countries lacking formal address systems can consider digital clinic-based systems such as those in SCALE to provide sufficiently precise spatiotemporal resolution of diagnosed TB patients to evaluate geographically-targeted ACF interventions from TB registration clinics. Extending these systems to TB testing, tracking positivity, and including questions on TB testing in Demographic Health Surveys would, first, allow underserved communities to be identified for ACF, ideally with simultaneous investment into strengthening routine services, and, secondly, provide guidance for when to stop.

Limitations of the study include the lower than anticipated prevalence, reduced intervention intensity (one instead of three rounds), insufficient power to detect a small difference in case notifications, and reduced data available due to censoring from March 2020 due to the impact of COVID-19. The intervention was only offered to those aged 18+ but the outcomes were assessed in those aged 15 and above which could lead to reduced observed impact, however, as TB prevalence increases with age in Malawi [17] any impact would have been minor. In addition, 25% of Blantyre-resident TB case-notifications during the relevant period had no co-ordinates recorded in our digital TB system (ePAL), a potential cause of ascertainment bias. It is also possible that the enhanced standard of care provided by the study clinic assistants at all primary facilities within Blantyre could have increased TB testing, and hence, caused the small increase in case notifications corresponding to our prevalence survey and intervention. We consider this unlikely, however, as facilities were staffed by study clinic assistants for more than 91 days before the start of the intervention in 56 of 72 clusters. Linkage to care and treatment was high though at 100% of those remaining within Blantyre City.

## Conclusions

Community-wide ACF can lead to substantial and rapid declines in TB burden following initial deployment in settings with high undiagnosed TB burdens, but well-implemented ACF interventions can fail to impact underlying TB epidemiology for a variety of reasons. Here we show evidence of diminishing returns and no remaining epidemiological impact from a previously effective ACF strategy in a high HIV prevalence city following several years of ACF and rapid declines in TB burden. In such settings, choices now lie between greatly increasing investment to provide highly sensitive screening to every individual or instead changing focus to targeted outreach and demand creation, alongside optimised facility-based and TB contact screening. Our data also show need for routine surveillance systems more attuned to rapidly changing TB epidemiology to meet TB elimination goals by 2035.

## Supporting information

S1 Text Including: Table A: Baseline characteristics identified through pre-intervention prevalence survey including original data. Table B: Clinical and microbiological characteristics of confirmed TB cases from pre-intervention prevalence survey. Table C: Demographic and microbiological characteristics of confirmed TB cases from ACF intervention.
(DOCX)

S1 File Trial protocol. (PDF)

S1 Checklist PloS Global Public Health questionnaire. (DOCX)

S2 Checklist CONSORT checklist. (DOCX)
