# Supplementary material for: Impact of active case-finding for tuberculosis on case-notifications in Blantyre, Malawi: A community-based cluster-randomised trial (SCALE)
Source: PLOS Glob Public Health. 2023 Dec 5;3(12):e0002683. doi: 10.1371/journal.pgph.0002683 (PMC10697577; doi:10.1371/journal.pgph.0002683)
Supplement: S1 Text — Including: Table A: Baseline characteristics identified through pre-intervention prevalence survey including original data. Table B: Clinical and microbiological characteristics of confirmed TB cases from pre-intervention prevalence survey. Table C: Demographic and microbiological characteristics of confirmed TB cases from ACF intervention. (DOCX) [file pgph.0002683.s001.docx]

**Table A: Baseline characteristics identified through pre-intervention prevalence survey including original data**

| Variable | Unit / category | ACF | | SOC | |
| --- | --- | --- | --- | --- | --- |
|  |  | % | n | % | n |
|  |  |  |  |  |  |
| Identified household members | |  | 10340 |  | 10559 |
| Sex | Male | 47.0 | 4779 | 46.4 | 4837 |
|  |  |  |  |  |  |
| Complete case individual survey participants | |  | 7620 |  | 7698 |
| HIV/ART status | HIV+ on ART  HIV+ not on ART  HIV unknown | 10.6  1.4  4.3 | 805 106 326 | 11.0  1.6  3.9 | 844  122  300 |
| TB contact (within 12 months) | Yes | 5.0 | 379 | 4.0 | 304 |
| Previous TB treatment | Yes | 2.7 | 206 | 2.8 | 217 |
| Reported TB symptoms | Cough (any duration)  Cough ≥ 2 weeks†  Night sweats  Weight loss  Fever  Any (cough any duration) | 3.5  2.0  5.3  5.6  3.2  13.7 | 264 153 405 425 240 1042 | 3.4  2.0  4.7  5.0  2.5  12.8 | 265 156  365 387  193  983 |

**Note:** Eligible household members were identified through an initial household survey. 15,897 of those eligible household members participated in the individual survey of which 13,318 had complete records.

**Table B: Clinical and microbiological characteristics of confirmed TB cases from pre-intervention prevalence survey**

| **Characteristics** | | | | **Symptom screening** | | | | | | **X-ray** | **Sputum results** | | | |
| --- | --- | --- | --- | --- | --- | --- | --- | --- | --- | --- | --- | --- | --- | --- |
| **Sex** | **Age** | **HIV status** | **Previous TB?** | **Cough** | **Chronic cough** | **Night sweats** | **Weight loss** | **Fever** | **Any TB symptoms** | **Chest X-ray** | **Smear** | **Xpert** | **Culture result** | **Culture ID** |
| Male | 45 | HIV negative | No | Yes | Yes | Yes | Yes | Yes | Yes | Abnormal | Negative | Positive | Negative | ND |
| Female | 32 | HIV positive | No | Yes | No | No | No | No | Yes | Normal | Negative | Negative | Positive | MTB |
| Female | 34 | HIV positive ART | No | Yes | Yes | Yes | Yes | No | Yes | Abnormal | Negative | Positive | Positive | MTB |
| Female | 29 | HIV negative | No | Yes | Yes | No | No | No | Yes | *-* | Negative | Positive | Positive | MTB |
| Male | 25 | HIV negative | No | Yes | Yes | No | No | No | Yes | Abnormal | Negative | Positive | Positive | MTB |
| Female | 19 | HIV negative | No | Yes | Yes | No | No | No | Yes | Abnormal | Positive | Positive | Positive | MTB |
| Female | 26 | HIV negative | No | Yes | Yes | No | No | Yes | Yes | Abnormal | Positive | Positive | Positive | MTB |
| Female | 22 | HIV negative | No | Yes | No | No | No | No | Yes | Normal | Negative | Negative | Positive | MTB |
| Male | 33 | HIV negative | Yes | Yes | No | Yes | No | Yes | Yes | Abnormal | Positive | Positive | Positive | MTB |
| Male | 19 | HIV negative | No | Yes | No | No | No | No | Yes | Abnormal | Negative | Negative | Positive | MTB |
| Male | 56 | HIV positive ART | No | Yes | Yes | No | No | Yes | Yes | Normal | Positive | Positive | Positive | MTB |
| Male | 45 | HIV positive ART | No | Yes | Yes | No | No | No | Yes | Abnormal | Negative | Positive | Negative | ND |
| Male | 40 | HIV negative | Yes | Yes | Yes | No | No | No | Yes | Normal | Positive | Positive | Positive | MTB |
| Female | 61 | HIV negative | No | No | No | No | No | No | No | Abnormal | Negative | Positive | Positive | MTB |
| Female | 89 | HIV negative | No | No | No | No | No | No | No | Abnormal | Negative | Positive | Negative | ND |
| Male | 27 | HIV negative | Yes | No | No | No | No | Yes | Yes | Abnormal | Negative | Positive | Contaminated | ND |
| Male | 27 | HIV negative | No | No | No | No | No | No | No | Abnormal | Negative | Negative | Positive | MTB |
| Male | 33 | HIV negative | Yes | No | No | No | No | No | No | Abnormal | Negative | Negative | Positive | MTB |
| Male | 43 | HIV negative | No | No | No | No | No | Yes | Yes | Abnormal | Negative | Positive | Positive | MTB |
| Male | 30 | HIV negative | No | No | No | Yes | No | No | Yes | Abnormal | Positive | Positive | Positive | MTB |
| Female | 38 | HIV negative | No | No | No | No | No | No | No | Abnormal | Negative | Negative | Positive | MTB |
| Male | 30 | HIV negative | No | No | No | Yes | No | Yes | Yes | Abnormal | Negative | Positive | Negative | ND |
| Female | 19 | HIV negative | No | No | No | No | No | No | No | *-* | Negative | Negative | Positive | MTB |
| Male | 44 | HIV negative | No | No | No | No | No | No | No | Normal | Positive | Positive | Positive | MTB |
| Male | 36 | HIV negative | No | No | No | Yes | No | No | Yes | Normal | Negative | Positive | Negative | ND |
| Male | 54 | HIV negative | No | No | No | No | No | No | No | Abnormal | Negative | Negative | Positive | MTB |
| Female | 37 | HIV positive ART | No | No | No | No | No | No | No | Abnormal | Negative | Positive | Positive | MTB |
| Male | 22 | HIV negative | No | No | No | No | No | No | No | Abnormal | Positive | Positive | Negative | ND |

**Table C: Demographic and microbiological characteristics of confirmed TB cases from ACF intervention**

| **Demographics** | | **Initial results** | | | | **Confirmatory results** | | | | |
| --- | --- | --- | --- | --- | --- | --- | --- | --- | --- | --- |
| **Sex** | **Age** | **Smear 1** | | **Smear 2** | | **Smear** | | **GeneXpert** | | **Culture** |
| Male | 47 | Negative | No AAFB | Positive | 1+ AAFB | Negative | No AAFB | Positive | Very low | MTB |
| Male | 38 | Positive | 3+ AAFB | Positive | 3+ AAFB | Positive | 3+ AAFB | Positive | High | MTB |
| Male | 37 | Positive | 3+ AAFB | Positive | 2+ AAFB | Positive | Scanty AAFB | Positive | Very low | MTB |
| Female | 42 | Positive | 3+ AAFB | Positive | 3+ AAFB | Positive | 3+ AAFB | Positive | Medium | MTB |
| Female | 56 | Positive | 3+ AAFB | Positive | 3+ AAFB | Positive | 3+ AAFB | Positive | High | MTB |
| Male | 26 | Positive | 3+ AAFB | Positive | 3+ AAFB | Positive | 1+ AAFB | Positive | Very low | MTB |
| Male | 53 | Positive | 3+ AAFB | - | - | Positive | 3+ AAFB | Positive | Medium | MTB |
| Male | 41 | Positive | 3+ AAFB | Positive | 3+ AAFB | Positive | 3+ AAFB | Positive | Medium | MTB |
| Male | 34 | Positive | Scanty AAFB | Positive | Scanty AAFB | Positive | Scanty AAFB | Positive | Low | MTB |
| Male | 35 | Negative | No AAFB | Positive | Scanty AAFB | Negative | No AAFB | Negative | Not detected | MTB |
| Female | 38 | Positive | 2+ AAFB | Positive | 2+ AAFB | Positive | 2+ AAFB | Positive | Medium | MTB |
| Male | 42 | Positive | 1+ AAFB | Positive | Scanty AAFB | Positive | 1+ AAFB | Positive | Low | MTB |
| Female | 31 | Positive | 3+ AAFB | Positive | 3+ AAFB | Positive | 3+ AAFB | Positive | Medium | MTB |

AAFB = Acid-alcohol-fast-bacilli , number (e.g. 3+) indicates number of bacilli seen

MTB=Mycobacterium Tuberculosis detected

Note: All ACF participants reported a cough of two weeks or more
