# Supplementary material for: Impact of active case-finding for tuberculosis on case-notifications in Blantyre, Malawi: A community-based cluster-randomised trial (SCALE)
Source: PLOS Glob Public Health. 2023 Dec 5;3(12):e0002683. doi: 10.1371/journal.pgph.0002683 (PMC10697577; doi:10.1371/journal.pgph.0002683)
Supplement: S1 File — (PDF) [file pgph.0002683.s002.pdf]

Sustainable **C**ommunity-wide **A**ctive Case Finding for **L**ung h**E**alth

Short title: SCALE

Research Organisation Ref: WT200901

Ethics Reference: 16228

Trial Registration Number: ISRCTN11400592

Date and Version No: Version 6.0, 13 November 2020

Principal Investigator: Prof Elizabeth Corbett, Dept. of Clinical Research,
London School of Hygiene & Tropical Medicine (LSHTM)
Keppel Street, London WC1E 7HT and Malawi Liverpool
Welcome Trust - Clinical Research Programme (MLW)
P.O. Box 30096 Chichiri, Blantyre 3, Malawi
Work +265 187 4628 or 187 6444 or 187 3871 Extension 303
Mobile +265 999 981 439 and +44 207 927 2360

Sponsor: London School of Hygiene & Tropical Medicine (LSHTM)
Keppel Street, London WC1E 7HT

Funder: Wellcome Trust.

Conflict of interests: We declare no conflict of interests

Compliance: The trial will be conducted in compliance with the protocol, ICH GCP Guidelines and other relevant regulatory requirements applying in the countries in which the trial will be conducted.

Confidentiality Statement

This document contains confidential information that must not be disclosed to anyone other than the Sponsor, the Investigator Team, host organisation, and members of the Research Ethics Committee, unless authorised to do so.

Principal Investigator Signature:

Date: **13 November 2020**

TABLE OF CONTENTS

1. ABBREVIATIONS 5
2. KEY TRIAL INVESTIGATORS AND CONTACTS 7
3. ABSTRACT 8
3.1. Background: 8
3.2. Study design: 8
3.3. Study site and participants: 8
3.4. Interventions 8
3.5. Outcomes: 8
3.6. Ethical issues: 8
3.7. Timelines: 9
3.8. Dissemination: 9
4. SYNOPSIS TABLE 10
5. BACKGROUND AND RATIONALE 13
5.1. Tuberculosis (TB) in high HIV prevalence settings 13
5.1.1. Burden of TB disease 13
5.1.2. Population-based active case-finding (ACF) 14
5.2. Study Rationale 15
5.2.1. Symptoms-plus-microscopy as an ACF strategy 15
5.2.2. Point-of-care TB and HIV diagnostics provided in the ACF intervention 16
6. RESEARCH QUESTIONS, AIMS AND OBJECTIVES 17
6.1. Research Questions 17
6.2. Specific aims 17
7. TRIAL DESIGN 18
7.1. Study site 19
7.1.1. Population-based clusters 19
7.1.2. Enhanced surveillance of routinely diagnosed TB patients in Blantyre 19
7.2. Study Population 20
7.3. Inclusion criteria 20
7.4. Exclusion criteria 21
7.5. Identification of participants and recruitment 21
8. RANDOMISATION AND BLINDING 22

8.1. Randomisation ... 22
8.2. Blinding ... 22
9. OUTCOMES AND OUTCOME EVALUATION ... 23
9.1. Primary and secondary outcomes ... 23
9.2. Additional pre-planned analyses ... 26
9.3. Case Definitions ... 26
9.3.1. Undiagnosed infectious (prevalent) TB ... 26
9.3.2. Other epidemiological definitions for prevalent TB ... 26
9.3.3. Routinely diagnosed TB patients ... 27
10. INTERVENTIONS, METHODS AND PROCEDURES ... 28
10.1. Recruitment ... 28
10.2. Screening and Eligibility Assessment ... 28
10.3. Informed Consent ... 28
10.4. Procedures ... 28
10.4.1. Pre-intervention survey participants ... 28
10.4.2. ACF intervention participants ... 29
10.4.3. Qualitative and economic sub-studies ... 30
10.5. Enhanced surveillance of TB in Blantyre District ... 30
10.5.1. Electronic TB database ... 30
10.6. Laboratory methods ... 31
10.6.1. ACF sputum specimens ... 31
10.6.2. TB sputum culture ... 31
10.6.3. Storage of specimens ... 31
10.6.4. Laboratory Safety ... 31
10.7. Discontinuation/Withdrawal of Participants ... 31
10.8. Definition of End of Trial ... 32
11. DATA COLLECTION AND MANAGEMENT ... 33
11.1. Data management ... 33
11.2. Data capture forms and questionnaires ... 33
11.3. Patient confidentiality ... 33
11.4. Data security ... 33
11.5. Quality assurance ... 33
12. ADVERSE EVENT MONITORING AND REPORTING ... 34
12.1. Anticipated harms from the intervention ... 34

12.2. Institutional responsibilities ... 34
12.3. Reporting Procedures ... 34
12.4. Good Clinical Practice (GCP) ... 35
12.5. Monitoring and audit ... 35
13. ECONOMIC EVALUATION ... 36
14. STATISTICS ... 38
14.1. Sample size justification ... 38
14.2. Data analysis plan ... 38
15. STUDY ADMINISTRATION AND INSTITUTIONAL RESPONSIBILITIES ... 39
15.1. Funding ... 39
15.2. Trial Sponsor and insurance ... 39
15.3. Trial Steering Committee ... 39
15.4. Data Safety and Monitoring Board ... 39
16. ETHICAL CONSIDERATIONS ... 40
16.1. Ethical Approvals ... 40
16.2. Informed consent at the different stages of the study ... 40
16.3. Participant compensation ... 40
16.4. Risks and risk-minimisation ... 40
16.5. Capacity-building ... 41
17. DISSEMINATION ... 42
17.1. Public engagement and scientific dissemination ... 42
17.2. Translating Research into Policy ... 42
18. STUDY TIMELINE ... 43
19. STUDY BUDGET ... 44
20. REFERENCES ... 45
APPENDIX 1 – PRE-PREVALENCE SURVEY TOOLS ... 48
APPENDIX 2 – ACF TOOLS ... 49
APPENDIX 3 – HEALTH FACILITY TB TESTING TOOLS ... 50
APPENDIX 4 – MATERIAL TRANSFER AGREEMENT ... 51

1. ABBREVIATIONS

AIDS	Acquired Immune Deficiency Syndrome
ACF	Active case-finding for TB
ART	Antiretroviral Therapy
BCG	Bacillus Calmette–Guérin vaccine
c-TB	Trade name (skin test for diagnosing latent tuberculosis infection)
CAD	Computer Assisted Diagnosis
CD4	Lymphocyte subset cell marker
CRF	Case Report Form
CTRG	Clinical Trials and Research Governance
CXR	Chest X-Ray
DHO	District Health Office
DSMB	Data Safety and Monitoring Board
EndTB	WHO strategy for TB elimination
ePAL	Electronic patient locator
GCP	Good Clinical Practice
GRADE	Grades of Recommendation Assessment, Development and Evaluation
HIV	Human Immunodeficiency Virus
ICF	Informed Consent Form
ICH	International Conference of Harmonisation
IRB	Independent Review Board
IUATLD	International Union Against Tuberculosis and Lung Disease
LAM	Lipoarabinomannan
LF	Lateral Flow
LSHTM	London School of Hygiene & Tropical Medicine
LTFU	Loss to follow-up
Mo	Month
MLW	Malawi-Liverpool-Wellcome Trust Centre
NTP	National TB Programme
OraQuick	Trade name (An oral HIV test kit)
PI	Principal Investigator
PLHIV	People living with HIV infection
REC	Research Ethics Committee

RSC	Research Support Center
SAE	Severe Adverse Event
SFTP	Secure File Transfer Protocol
SOC	Standard of Care
SOP	Standard Operating Procedure
TB	Tuberculosis
TSC	Trial Steering Committee
TST	Tuberculin Skin Test
WHO	World Health Organisation
Xpert	Trade name: (automated nucleic acid amplification TB test: MTB/RIF or Ultra)
ZAMSTAR	Zambia South Africa TB and HIV Reduction study

2. KEY TRIAL INVESTIGATORS AND CONTACTS

Role	Name	Institution	Contact details
Principal Investigator	Prof Liz Corbett	London School of Hygiene & Tropical Medicine	liz.corbett@lshtm.ac.uk or lizcorbett04@gmail.com<br>+44 207 927 2360<br>+265 999 981439
Co-Investigator / and Trial Statistician	Prof Katherine Fielding	London School of Hygiene & Trop Med	katherine.fielding@lshtm.ac.uk
Co-Investigator / Economics	Dr Hendramoorthy Maheswaran	University of Liverpool	H.Maheswaran2 @liverpool.ac.uk
Co-Investigator / Epidemiology	Dr Peter MacPherson	Liverpool School of Trop Med	Peter.MacPherson @lstmed.ac.uk
Co-Investigator / Qualitative	Dr Nicola Desmond	Liverpool School of Trop Med	Nicola.desmond@lstmed.ac.uk
Co-Investigator / Mathematical modelling	Dr Peter Dodd	University of Sheffield	p.j.dodd@sheffield.ac.uk
Co-Investigator / Malawi Ministry of Health	Dr James Mpunga	National TB Control Programme Manager	mpungajay@gmail.com
Trial Coordinator	Helena Feasey	London School of Hyg & Trop Med	Helena.feasey@lshtm.ac.uk
Data Manager	Lingston Chiume	Malawi Liverpool Wellcome (MLW)	chiume.ling@gmail.com<br>lchiume@mlw.mw
Field site Coordinator	George Sinjani	Malawi Liverpool Wellcome (MLW)	geosinjani@gmail.com<br>gsinjani@mlw.mw
Sponsor	Clinical Trials Sub-Committee	London School of Hygiene & Tropical Medicine	ctu@lshtm.ac.uk
Trial steering Committee (TSC)	Jacob Creswell	StopTB Partnership Geneva	jacobc@stoptb.org
Trial steering Committee (TSC)	Prof Ibrahim Abubakar	University College, London	i.abubakar@ucl.ac.uk
Trial steering Committee (TSC)	Prof Peter Godfrey-Faussett	Unaids, Geneva	FaussettP@unaids.org

3. ABSTRACT

3.1. Background:

Tuberculosis (TB) is the leading infectious cause of death, globally, with 1.6 million deaths estimated for 2017. The United Nations have endorsed an ambitious plan to find 40 million undiagnosed TB cases by 2022 as part of the WHO EndTB strategy. Active case finding (ACF) In the community was a widely used strategy in the 20th Century, and is part of the NTP strategy. However, the broader benefits of ACF on underlying TB epidemiology remain unclear and limit the willingness to implement this strategy widely in urban setting such as Blantyre, Malawi, where about 1% of adults had undiagnosed TB in 2013-14. Patients with TB symptoms have a high risk of underlying HIV.

3.2. Study design:

A single-site (Blantyre, Malawi) cluster-randomised clinical trial with two study arms (1:1 recruitment) covering a population of ~320,000 adults.

3.3. Study site and participants:

Participants in 72 clusters of Blantyre city will be recruited to the two-phase study:

- pre-intervention TB and HIV prevalence survey of 15,500 adults (18 years or older)
- a 1 year ACF intervention providing 1 round any given cluster

3.4. Interventions

Prevalence Survey: TB screening will be based on digital chest x-ray read by an experienced radiographer assisted by computer assisted diagnostics. Patients reporting chronic cough or with abnormal chest x-ray will be asked to submit 2 sputum specimens for TB testing with Xpert (automated nucleic acid amplification test), microscopy and culture. Oral HIV testing will be offered to all participants in the pre-intervention survey. Finger prick rapid HIV diagnostic tests will be used to confirm positive results, and will be offered to all participants.

ACF intervention: All symptomatic household members identified on brief door-to-door enquiry for chronic cough will be left information leaflets, sputum collection pots, with sputum specimens collected the following day for microscopy. Results will be reported within 2 to 4 days. ACF participants will also be offered an oral HIV self-testing kit with instructions on use if HIV status is unknown and, if HIV positive, will be offered confirmatory testing.

3.5. Outcomes:

Primary: to compare between randomisation arms the rate of treatment for microbiologically-confirmed TB (per 1000 adult residents per year) during 3 months starting from the first ACF intervention.

3.6. Ethical issues:

The intervention has minimal potential for harm, with all components already conforming to International Best Practice: rather the research component relates to the impact evaluation through prevalence survey and skin testing of a public health intervention. Written or witnessed informed consent will be taken from all survey.

Written or witnessed informed consent will be taken from all ACF participants. We request adaptation of informed consent procedures for the ACF HIV self-testing, using verbal consent documented by unnamed thumbprint instead as this conforms to Malawi National Policy and the HIV Prevention and Management Act.

Participants with TB disease, or newly diagnosed HIV will be counselled and referred for treatment.

3.7. Timelines:
3 year study from Sept 2018 to Aug 2021, with the ACF Intervention and all surveys delivered during June 2019 to March 2020.

3.8. Dissemination:
Regular meetings will be held with Blantyre District Health Office and the National TB Programme. The Principal Investigator of SCALE has already had substantial influence on International Policy and Practice for TB screening and ACF through the 2013 WHO Guidelines, and on National TB Programme Policy in Malawi through the 2016 Malawi National TB Research Network. She is well connected to the Malawi Ministry via both HIV and TB Technical Working Groups. The National TB Programme Manager is a co-investigator on this proposal.

Dissemination meetings will be held with cluster residents, National TB programme and Blantyre District health office on completion of the project. Study findings will be reported through conference presentations and peer-reviewed publications.

4. SYNOPSIS TABLE

Trial Title	**S**ustainable **C**ommunity-wide **A**ctive Case Finding for **L**ung h**E**alth
Short title	SCALE
Trial Design	A single-site (Blantyre, Malawi) cluster-randomised clinical trial with two study arms (1:1 recruitment)
Trial Participants	1. **All residents** in defined study neighbourhoods of Blantyre, Malawi, will **be enumerated.** Adults (≥18 years) will be **monitored for TB investigations and diagnoses** using an enhanced surveillance system delivered by routine Blantyre District TB Officers<br><br>2. A **random sample of households** from which all adult members will be invited for TB and HIV screening (pre-intervention survey)<br>a. Radiological and symptom screening<br>b. Confirmatory TB testing nucleic acid amplification testing and culture<br><br>3. **All households in clusters randomised to the active case-finding (ACF)-arm** will have **1 brief enquiry for prolonged cough** among household members **over 10-11 months.**<br><br>Reported coughers aged ≥18 years will be offered:<br>• investigation for TB using two sputum samples for sputum-microscopy, with results reported back at cluster-level, and<br>• provider-initiated HIV self-testing using OraQuick<br><br>Linkage to confirmatory testing and TB and HIV treatment will be facilitated for all participants testing TB and/or HIV positive
Planned Sample Size	72 clusters (each of ~4,400 adult residents) with boundaries defined by community health worker (CHW) catchment areas, plus all 15 health facilities providing TB Registration for Blantyre City residents.<br><br>• Total population ~320 thousand adults (~160 thousand adults/arm) will be included in the study area, for which brief household enumeration will be conducted in collaboration with Blantyre DHO.<br><br>• In collaboration with Blantyre DHO geolocation and national TB programme data are already collected on all (~2,500 per year) TB patients. These records will be used to assign cluster-resident status (yes/no; cluster number and trial arm) during this trial<br><br>• ACF arm participants: we anticipate up to 10,000 adults (~2% adults per 6-month household enquiry for cough) will be identified by the ACF intervention as needing investigation for TB and HIV<br><br>• Pre-intervention prevalence survey participants: residents from both intervention and non-intervention clusters will be invited for

	TB screening, with HIV testing offered to all participants: 15,480 adults (215 per cluster)<br><br>• Qualitative and economic sub-studies will recruit up to 100 participants being investigated for TB, having reported prolonged cough to the TB ACF outreach team or primary care facilities. Serial in-depth interviews will be used to investigate motivations for health-seeking, and the social and economic consequences of TB investigations and diagnosis.	
Follow up duration	1-3 months per cluster<br><br>Pre-intervention survey, 10-11 months of ACF intervention: cluster-initiation will be staggered over 9-months	
Planned recruitment period	June 2019-Mar 2020: pre-intervention survey and ACF Round 1	
	Objectives	Outcome Measures/Endpoints
1 Primary Outcome	To determine the impact of TB-ACF on population-level:<br><br>2.1 Rates of adult registration for microbiologically-confirmed TB	To compare between randomisation arms:<br><br>1.1 The rate of treatment (per 1000 adult residents per year) during the 3 months starting from the first ACF intervention, defined by entry into the District Health Office TB Treatment Register and laboratory records
2 Other pre-planned analyses	To investigate the impact of TB-ACF on population-level:<br><br>2.1 Demand for routine TB testing services<br><br>To determine the impact of **provider-initiated HIV self-testing** for TB-ACF participants on:<br><br>2.2 Numbers of newly diagnosed HIV-positive participants, and numbers subsequently linking into HIV care	To compare **between randomisation arms**:<br><br>3.1 The rate (per 1000 adult cluster residents p.a.) of investigation for TB, defined by entry into the "Presumptive TB Register" in selected routine TB Registration Facilities<br><br>**Within clusters randomised to the ACF-arm only**, to document rates (per 1000 adult cluster residents p.a.) of:<br><br>3.2 New diagnosis of HIV made by the ACF team using OraQuick, with follow-up for confirmatory testing and ART initiation

	2.3 Numbers of participants diagnosed with TB using sputum microscopy	3.3 New diagnosis of TB made by the ACF team using microscopy
4. Economic and mathematical modelling analyses	4.1 to determine the costs<br>• per adult screened with microscopy during ACF<br>• per smear+ve TB patient detected by the ACF team<br>• per additional adult investigated for TB by the routine health system<br>• per additional adult diagnosed with TB by the routine health system<br>• likely final scale-up programme costs<br><br>4.2 to determine the incremental costs of adding HIV to TB ACF<br>• per adult screened for HIV by the ACF team<br>• per adult with HIV detected by the ACF team<br>• per adult started on ART<br><br>4.3 to estimate the cost-effectiveness per new TB infection, TB case, and TB death averted by TB and TB+HIV ACF	4.1 Costing of ACF from the provider-perspective. Denominators will use records from the ACF team, and routine health system data collected by trial arm under the extended TB surveillance system.<br><br>4.2 Costing of HIV self-testing HIV-positive clients to the TB-ACF, with denominators obtained from ACF team and facility records<br><br>4.3 Fully costed mathematical modelling of the TB-ACF intervention with and without additional HIV testing components.
5. Transmission modelling analyses	5.1 To determine the fraction of Blantyre TB transmission attributable to health facility and HIV care clinic exposure<br>5.2 To investigate the potential epidemiological impact of spatially-targeted TB control interventions and improve infection control and prevention at health facilities and HIV care clinics	5.1 Transmission modelling of genomic and TB epidemiological data
Investigational Trial Intervention(s)	Public health trial investigating the impact on underlying TB epidemiology of international best practice interventions: no investigational interventions.<br><br>TB messaging and leafleting, with community-level TB and HIV diagnosis provided for all adult residents in intervention-arm clusters through periodic door-to-door outreach ACF symptom screening plus (if symptomatic): TB microscopy of sputum, HIV diagnosis using oral OraQuick HIV-1/2 packaged to allow the option of HIV self-testing.	

5. BACKGROUND AND RATIONALE

5.1. Tuberculosis (TB) in high HIV prevalence settings

5.1.1. Burden of TB disease

TB has been the leading cause of adult deaths from an infectious disease, globally, since 2011, with an estimated 10.0 million new TB cases and 1.6 million TB deaths, including 900 thousand cases and 300 thousand deaths in people living with HIV (PLHIV), in 2017 (1). HIV infection greatly increases the risk of progression from TB infection to TB disease, and also increases the risk of death when TB disease does occur. Because of the regional magnitude of the HIV epidemic, Southern Africa has the highest TB incidence of any global region, with an estimated 84% of all HIV-related TB deaths, globally (1,2).

Prospects for TB control in Africa have changed dramatically in the last 15 years with the highly successful scale-up of antiretroviral (ART) services and global health initiatives for funding TB and HIV programmes (2,3). However, there is still no accurate but low-cost TB test that can be reliably delivered at the point-of-care to diagnose pulmonary disease (4-5). Accessing TB diagnosis remains highly problematic, notably so for adult men who account for most TB cases and deaths but are not as well served as women by TB or HIV programmes (1,2,5-8). This maintains high prevalence of undiagnosed TB (>1% prevalence in urban Blantyre in 2013-14), and hence transmission rates, within communities. Of note, most transmission events occur to casual contacts, limiting the effectiveness of contact tracing strategies, and most TB in high transmission settings results from recently acquired TB infection (9).

Without control of ongoing TB transmission, the full potential of ART and preventive therapy for HIV-positive individuals may not be realized for decades: rapid declines in TB incidence in this region require combined prevention strategies including improved case-finding and treatment (10,11). In Malawi, for example, smear-positive TB case-notifications declined by 16% over a 12 year period when ART coverage (proportion of HIV-infected people retained on ART) increased from <0.5% to 41%, and have declined further since then (12) but are unlikely to the WHO EndTB strategy targets endorsed for 2022 and 2030 unless the large TB-testing" gap can be reduced, including for men (13-15).

Recent TB prevalence surveys including 11 African countries, provide the first regionally representative data since the 1960s (16,17). These confirm the same key findings first reported from subnational surveys in South Africa and Zimbabwe (18,19). Meta-analysis of 86 surveys with 3.1 million participants shows (16): -

- much higher than anticipated rates of undiagnosed infectious TB --- the main driver of TB transmission --- prompting 2 upward revisions of WHO Global TB estimates
- men and urban residents at disproportionately high risk of undiagnosed TB
- prolonged infectiousness before diagnosis or death (median 2.6 years for men and 1.6 years for women) as deduced from comparison with case-notification data
- most survey participants with undiagnosed infectious TB being HIV-negative, even in African countries.

In Malawi and neighbouring Zambia, 2013/14 prevalence surveys report 901 and 993 culture-positive participants per 100,000 urban adults screened, respectively (16), with approximately 2.4 undiagnosed TB cases for every case diagnosed in any given year in Malawi. However, the pre-intervention prevalence survey conducted as the first phase of this study has initial

results showing only 200 confirmed TB positive participants per 100,000 urban adults screened.

5.1.2. Population-based active case-finding (ACF)

At high prevalence, population-based active case-finding (ACF) for TB has potential to rapidly improve control and reduce TB transmission rates and can even be cost-saving if a sufficiently high efficiency of case-finding can be delivered at costs below a threshold proportional to the costs of treating TB (20). Recognising the importance of undiagnosed TB, but also the potential waste of resources and harms from poorly-conducted interventions, the 2013 WHO TB Systematic Screening Guidelines provide conditional support for ACF interventions in geographically defined communities with a prevalence of undiagnosed TB of 1% or more (5).

Population-based ACF was used extensively in the Northern Hemisphere until the 1970s based on radiology screening algorithms that, although highly sensitive, requires capital investment and skilled operators not readily available in low-income countries (5,21,22). Significant reductions in the prevalence of undiagnosed TB were also reported more recently from Vietnam, in a cluster-randomised trial that conducted annual collection of sputum for Xpert MTB/RIF regardless of symptoms from all adult residents for 4 successive years (23).

A less costly ACF intervention has been used in several African countries, based on encouraging participants to volunteer chronic cough to outreach or door-to-door teams for investigation with smear-microscopy for TB diagnosis. In Harare, Zimbabwe, estimated per-round efficiency was 20-25% (proportion of all undiagnosed cases found) (24), but population-level smear-positive TB case-notifications were doubled and accompanied by rapid and substantial reductions (before-to-after unadjusted -43%; adjusted -41%) in undiagnosed infectious TB from 5 rounds of ACF delivered over 2.5 years (24). Although less robust than a randomized design, this magnitude of effect within a short timeframe still meets criteria for likely causality (22).

Introducing the Harare model (ACF using 6-monthly enquiry for chronic cough, investigated using smear-microscopy) to high-density neighbourhoods in Blantyre, Malawi, during 2012-14 doubled smear-positive TB case-notification rates with a similar time-trend as seen in Harare. A similar effect has been reported from interventions using periodic direct enquiry for chronic cough with sputum collection in the community in rural Ethiopia and urban Brazil (25,26).

Mathematical modelling suggests a likely pronounced effect on underlying TB epidemiology, with a projected marked decline in undiagnosed TB prevalence (as in Zimbabwe and Vietnam) and new incident TB cases. However, ACF-interventions have not yet been accompanied by the anticipated “accelerated decline” in TB case-notification rates post-ACF intervention (17). TB case-notification data are a complex mix of disease episodes, with any given case reflecting TB transmission that could have occurred anywhere between a few weeks to many years ago.

In the absence of a prevalence survey data in Malawi, we investigated whether or not there may have been a relatively long-lasting behaviour-change --- whereby individuals who have interacted with the ACF team may have been “behaviourally primed” to act more promptly when they next develop TB symptoms. Behavioural priming is thought to have contributed to the success of a household case-finding study in Zambia and South Africa (as a larger effect was seen than could be explained by TB cases found or prevented), and also provided a better fit to a mathematical model of the Blantyre ACF data than could be obtained without

making this assumption. An initial pilot study in Blantyre has provided data to support long-lasting behaviour-change, with lasting recall of the ACF intervention and its key messages demonstrated by community-members 2 years after the last round of ACF.

5.2. Study Rationale

With ACF now becoming mainstream global policy, there is a pressing need to definitively investigate the more promising approaches to community-wide ACF, where costs but also potential benefits are high, and where contemporary examples with impact evaluation have given conflicting results (21,23-27).

With four separate studies showing evidence of population-level effect (21,24-26) the Harare-ACF approach described above already has a relatively strong evidence-base, but has still not been widely implemented in Africa. In part, this reflects the "conditional recommendation" for this type of intervention from the 2013 WHO Guidelines for Systematic Screening for active TB (5), graded as such because of weak evidence at that time:

- no cluster-randomised trials with population-level endpoints had been carried out to evaluate the Harare-ACF intervention (randomised trials are required to qualify as "Strong Evidence" under the GRADE system)
- the only case-finding intervention investigated with a cluster-randomised trial using prevalence survey/TB transmission endpoints (ZAMSTAR) evaluated 2 different interventions in a multifactorial design and found no benefit in the general population enhanced case-finding (ECF) arm, and a relatively small and marginally-significant effect (p=0.06) in the "household-contact" arm (27). Unlike the Harare/Blantyre-ACF intervention, no "peak" in TB case-notifications was reported in the ECF arm, suggesting that the intervention (delivered indirectly through schools, facilities, and community hubs such as market places) was either not effective, or did not reach the necessary intensity/coverage to have efficacy at population level.

A cluster-randomised trial result showing an increase in TB case-notifications from a relatively low-cost and replicable ACF strategy would contribute to regional ACF practice, increasing willingness to invest in broader implementation and impact evaluation (22, 28).

5.2.1. Symptoms-plus-microscopy as an ACF strategy

The ACF strategy has notable limitations (29), including low sensitivity, difficulty producing sputum, and prevalence survey findings that only ~50% of individuals with undiagnosed TB in the community report chronic cough. In Harare, only an estimated 20% to 25% of undiagnosed TB cases were found in any given ACF round (21).

Nonetheless, this was sufficient to double case-notifications and substantially reduce undiagnosed TB, with a similar estimated magnitude to for annual Xpert-based screening in Vietnam. Of note, the more pronounced the contribution of behaviour-change component of ACF --- which can contribute more than direct ACF diagnosis in the field (24,30) --- the less sensitive the strategy will be to field-diagnostic performance (31).

Advantages of smear-microscopy are high throughput, low consumable costs (~$USD 0.50 in Blantyre compared to ~$23 for Xpert MTB/RIF), low equipment costs, and easy supply chain management and maintenance. Microscopy is still far the most widely used bacteriological test for TB, globally, and also in Malawi. The National TB Programme already supports highly decentralised smear-microscopy, has community collection-points, and has included community-based ACF in urban slums as part of the current NTP strategy.

The other feasible diagnostic strategy would be chest radiography (X-ray), which has high sensitivity and can be combined with a specific confirmatory test, such as Xpert, with or without use of preliminary symptom screening (5,29). Radiography for TB screening is becoming more feasible for low resource settings, with digital images, falling hardware price of, and computer-assisted diagnostics (CAD) that perform as well as expert readers (32,33). Population-based radiological screening is still a major undertaking, requiring substantial capital investment and dependent on adequate road networks (34). Moreover, in Malawi, radiography has not been fully decentralised below District Hospital level, making community-based screening incongruous at the present time (5).

5.2.2. Point-of-care TB and HIV diagnostics provided in the ACF intervention

HIV counselling and testing: ACF participants will be offered **oral HIV test kits, with the option of self-testing**. This recognises the high prevalence of undiagnosed or untreated HIV in community-members with cough (35), and the high acceptability, safety and accuracy of community-based HIV self-testing (8,36,37). ACF team members will be trained to support accurate HIV self-testing and to provide brief health information and post-test counselling following an approach developed and optimised in Malawi, and now adopted as part of national HIV programme policy.

For SCALE, ACF participants will be offered the choice of **HIV self-testing (OraQuick® HIV Self-Test)** either at an ACF tent located within the intervention cluster, or at home. Participants self-testing HIV-positive will have confirmatory testing using finger-prick rapid HIV test kits.

Newly diagnosed or untreated PLHIV will be referred to routine services for ART (available at primary care level with same-day initiation in Blantyre).

6. RESEARCH QUESTIONS, AIMS AND OBJECTIVES

The **broad objectives** are to investigate whether providing periodic TB/HIV ACF, focused on investigation of chronic cough leads to increased TB case-notifications.

6.1. Research Questions

The key research questions are: -

1) Does community-wide ACF increase the rate of diagnosis of microbiologically-confirmed TB at primary care clinics? If so
   - With what time-course relative to the ACF visit?
   - Are time trends the same for microbiologically-confirmed and microbiologically-unconfirmed TB?
   - To what extent does increased case-notifications relate to increased demand for TB testing services, rather than direct diagnosis by specimens submitted to the ACF team?
   - In the context of declining case notification rates, what fraction of Blantyre TB transmission is attributable to health facility and HIV care clinic exposure?

2) Is delivery of HIV self-testing alongside the offer of TB testing acceptable to symptomatic ACF participants of unknown HIV status?
   - What is the diagnostic yield (number of confirmed new HIV-positive individuals per 100 self-testers) from HIV self-testing provided alongside ACF for TB?

6.2. Specific aims

The specific aims are to:

1. To estimate the pre-intervention prevalence of undiagnosed TB in Blantyre, using combined radiological and bacteriological survey of 15,500 adults.
2. To carry out a cluster-randomised trial with 72 clusters (~4,400 adults each) randomised to: enhanced Standard of Care (SOC) arm or intervention (ACF) arm
3. To compare between randomisation arms after 1 round of ACF:-
   a. Time trends in microbiologically-confirmed TB case notification rates
4. To investigate the broader effect of ACF on health-seeking behaviour
5. To investigate the costs, cost effectiveness, and projected impact of ACF for TB and HIV on underlying TB epidemiology
6. To investigate the fraction of Blantyre TB transmission attributable to health centre and HIV care clinic exposure

7. TRIAL DESIGN

SCALE is a cluster-randomised trial of TB/HIV active case-finding with constrained randomization (1:1 ratio) of 72 clusters of ~4,400 adults per cluster, with boundaries defined by community health worker catchment areas

Figure 1. Original study design.

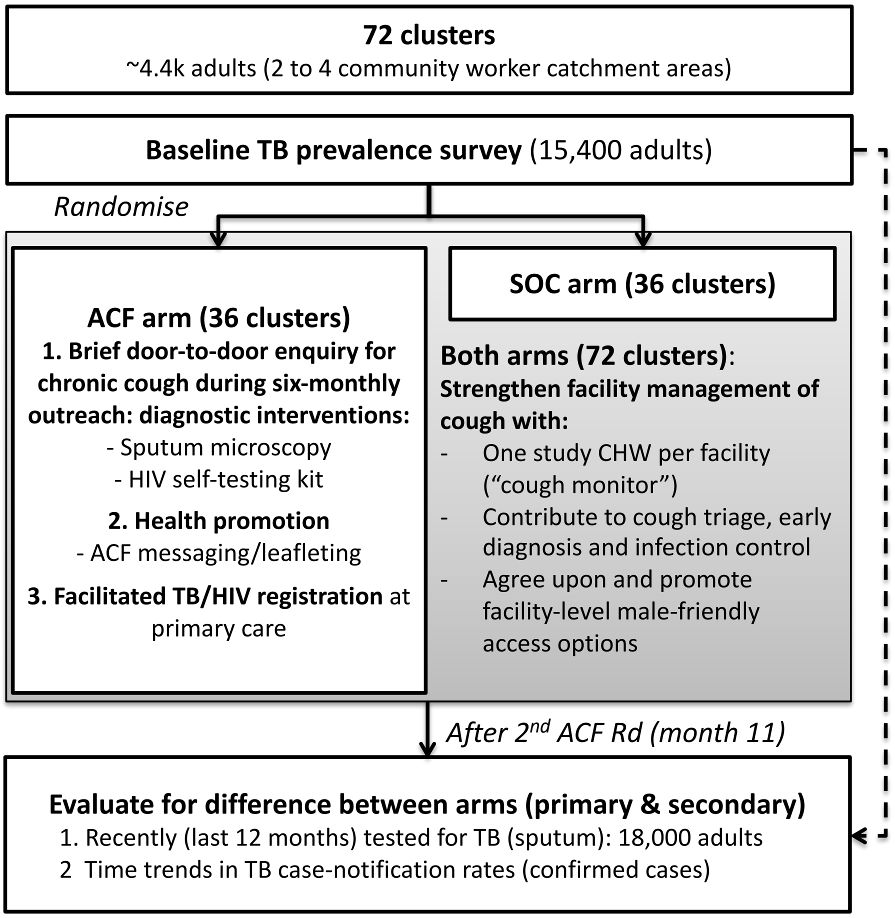

Previously undefined abbreviations: SOC= Standard of Care; k= thousand; mos= months

Clusters randomised to the ACF intervention arm will receive 1 round of brief door-to-door enquiry for chronic cough, repeated after 10 months, with staggered cluster initiation over 9 months. Health promotion messaging will be provided at the time of ACF, through leafleting and door-to-door interactions at the time of brief enquiry for chronic cough.

All clusters (Enhanced Standard of Care): Irrespective of randomisation, each facility in the study area will be provided with one study staff member (community/environmental health worker) responsible for facilitating identification of attendees with cough for TB investigations and basic infection control; receiving TB or HIV positive clients referred following ACF; and assisting District TB Officers to register patients being investigated for TB, with a special focus on men.

Outcomes will be evaluated for months 1-3 from ACF initiation using routine case-notification data for TB case-notification rates.

STUDY SITE AND POPULATION

7.1. Study site

7.1.1. Population-based clusters

SCALE will be implemented in the geographically-defined catchment population of 315 community health workers (CHW) in the high-density and peri-urban residential areas of Blantyre City. CHW catchment areas have been grouped to form 72 clusters with a population of ~4,400 adult residents each (~320,000 adults total). CHW catchment areas and study population was defined by a city-wide brief enumeration with Blantyre DHO in 2015.

The study population is served by 11 Government Primary Care clinics (Bangwe, Chileka, Chilomoni, Limbe, Makhetha, Mbayani, Ndirande, Soche Maternity, South Lunzu, Zingwangwa). Adult HIV prevalence is ~18% and the prevalence of active tuberculosis ~1,000 per 100,000 adults. On-site TB diagnostics available through the routine clinic services include sputum microscopy, with some clinics also having Xpert MTB/Rif and digital chest radiography. All clinics offer point-of-care HIV testing, and immediate ART under the Malawi National Guidelines.

Figure: Satellite map image of proposed SCALE clusters

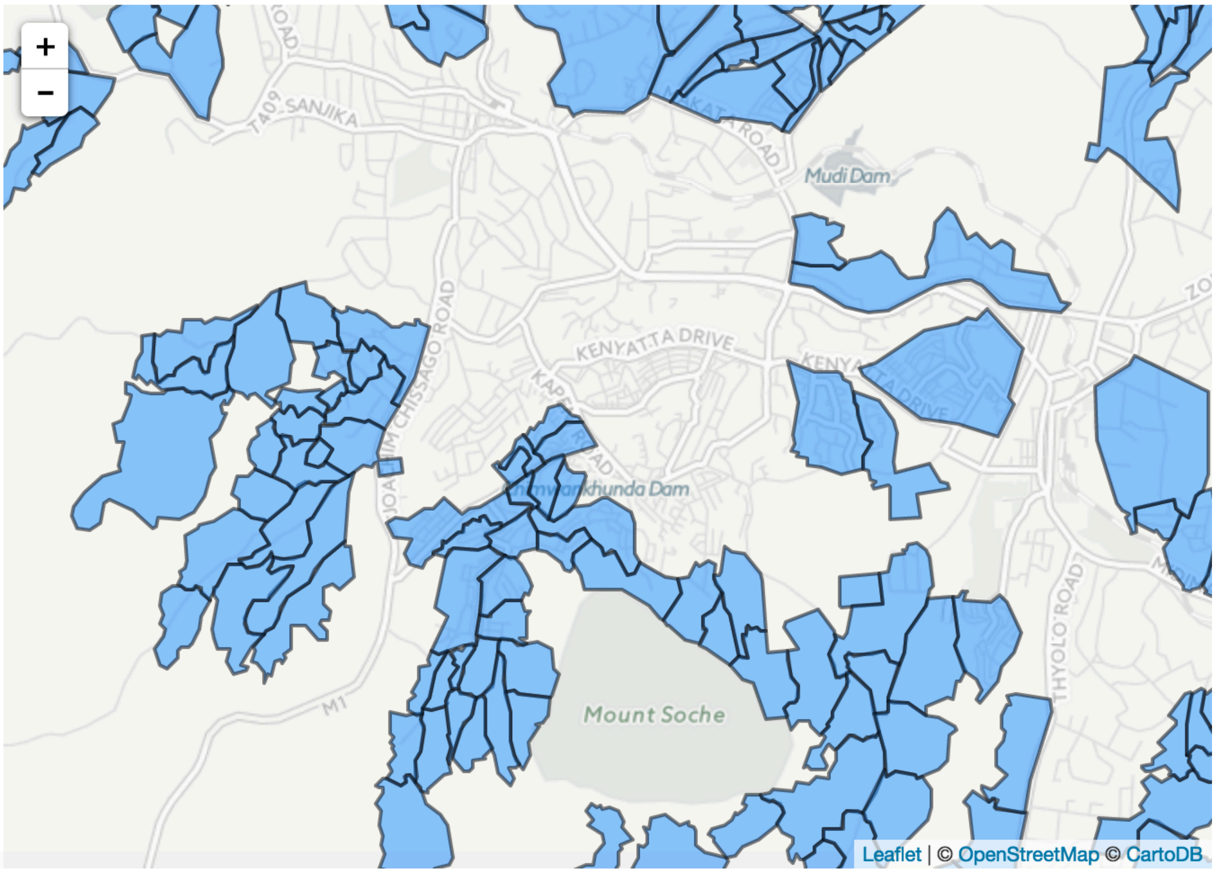

7.1.2. Enhanced surveillance of routinely diagnosed TB patients in Blantyre

In collaboration with Blantyre District Health Office (DHO), place of residence (GPS coordinates) and national TB programme data will be collected on all (~2,500 per year) TB

patients into an electronic database, together with a sputum specimen for TB microscopy and culture.

These Electronic TB Register records will be used to assign cluster-resident status (yes/no; cluster number and trial arm) for all registering TB patients during this trial, allowing cluster-level TB case-notification rates to be calculated.

For the purposes of this protocol, MLW will work with Blantyre DHO to adapt the current paper-based Presumptive TB Register, used to record patients being investigated for TB and the results of microbiological tests, into an Electronic Register that allows place of residence GPS coordinates to be captured, enabling investigation of cluster-level rates of TB investigations at primary care level (Appendix 4.3a&b Consent, Appendix 4.5a&b Patient Information Sheet and Appendix 4.4 CRF). MLW will also work with Chiradzulu DHO to add Chiradzulu District Hospital to the Electronic Register, since it has been identified that approximately 34% of patients who registered for TB treatment there in 2017-2019 came from the Blantyre urban area.

Qualitative in-depth interviews will be carried out with purposively selected patients being investigated for TB at primary care level because of cough, aiming to identify cues to action (Appendix 4.1a&b Consent forms, Appendix 4.6a&b participant information sheets and Appendix 4.8 topic guide).

7.2. Study Population

Different populations will be selected for the 2 main phases of the trial (pre-intervention survey and an intervention phase).

The following participants will be recruited for the different stages of the study.

- 15,500 adults selected for the pre-intervention TB and HIV survey
- An estimated 10,000 symptomatic ACF intervention participants

7.3. Inclusion criteria

Inclusion criteria for the pre-intervention **prevalence survey**: -

- Aged 18 years or older
- Resident of an intervention or non-intervention cluster
- Able and willing to provide written (or witnessed) informed consent
- Intending to remain within Blantyre City for at least the next 8 weeks
- Willing to have radiological screening for TB (with abdominal screening for women of child bearing age)

Inclusion criteria for the ACF intervention: -

- Aged 18 years or older
- Resident of an intervention cluster
- Reported cough for 2 weeks or longer
- Able and willing to provide written (or witnessed) informed consent
- Willing to provide sputum for TB testing

7.4. Exclusion criteria

Exclusion criteria for the ACF intervention

- Currently on TB treatment

7.5. Identification of participants and recruitment

Pre-intervention prevalence survey: participants will be recruited from their households, following a preliminary visit by a research assistant to explain the purpose of the study and ask for permission to book a convenient appointment for informed consent and study procedures.

All investigations will be carried out within the community, with no need to travel unless the participant is diagnosed with HIV, TB, or has an abnormal chest radiograph that requires referral for further investigations or treatment.

Pre-intervention survey participants will be recruited using random sampling based on satellite-map images.

Participants (or the Parents/Legal Guardians of participating children) meeting inclusion criteria will be provided with oral and written information about the study by the research assistant (Appendix 3.12a&b flyer and Appendix 3.14a&b information sheet) and will be invited to provide written informed consent to participate in the study (Appendix 3.4a&b consent form). Participants who are illiterate will be asked to provide a thumbprint confirmation of consent, which will be observed and recorded by a witness independent of the study team. Completed consent forms will be securely stored as trial source documents within a trial folder.

ACF intervention participants: participants will be recruited from their households, following brief door-to-door enquiry for chronic cough among any household member. Participants will be provided with oral and written information about the study by the research assistant (Appendix 2.1a&b information sheet and consent form) and will be invited to provide written informed consent to participate in the study. Participants who are illiterate will be asked to provide a thumbprint confirmation of consent, which will be observed and recorded by a witness independent of the study team. For participants with reported cough who are not present, the information sheet and consent form (Appendix 2.1a&b) will be left for them to read/have read to them and complete the form for return to the research assistant, along with the sputum pots.

For community-based HIV self-testing, which (unlike sputum collection) is being provided face-to-face, oral consent documented by a thumbprint will be used, in line with national policy and legislation (Appendix 2.2 a&b oral consent and Appendix 2.3 a&b thumbprint consent form).

8. RANDOMISATION AND BLINDING

8.1. Randomisation

The unit of randomisation is the cluster.

Randomisation (1:1 by arm) will be conducted at a public meeting, using random selection of one number from a previously prepared list of 999 randomly selected allocations generated by the trial statistician using a computer programme.

Randomisation will be constrained to avoid extreme differences in geographical distribution between the two arms, and to provide balance in the following variables: -

- Mean distance from cluster centres to the nearest health clinic
- TB case-notification rates as captured by the Blantyre District TB Officers
- Clusters that have received ACF in a previous study.

8.2. Blinding

Because of the nature of the study and the interventions offered, it will not be possible to blind participants or research assistants to allocation groups.

Nevertheless, steps will be taken to ensure that the investigators, including the Principal Investigator and Trial Statistician will remain blinded to allocation groups until database lock preceding final analysis. No unblinded interim analysis will be conducted.

9. OUTCOMES AND OUTCOME EVALUATION

Evaluation of the primary outcome will use data from: -

- Blantyre District Electronic TB Register (captured by routine District Health Office staff)

9.1. Primary and secondary outcomes

The **primary outcome** will compare between arms

1. **The rate of treatment for microbiologically-confirmed TB (per 1000 adult residents per year) during 3 months starting from the first the ACF intervention**, defined by entry into the District Health Office Electronic TB Treatment Register and laboratory records, with cluster resident status of registering TB patients defined by Global Positioning System (GPS) coordinates for place of residence captured by a satellite map application (ePAL) in routine use by Blantyre District TB Officers.

Table of objectives, outcomes and pre-planned analyses

SCALE	Objectives	Outcome Measures/Endpoints
1. Primary Outcome	To determine the impact of TB-ACF on population-level:<br><br>2.1 Rates of adult registration for microbiologically-confirmed TB	To compare between randomisation arms:<br><br>2.1 The rate of treatment (per 1000 adult residents per year) during the 12 months starting from the first ACF intervention, defined by entry into the District Health Office TB Treatment Register and laboratory records
3 Other pre-planned analyses	To investigate the impact of TB-ACF on population-level:<br><br>3.1 Demand for routine TB testing services<br><br>To determine the impact of **provider-initiated HIV self-testing** for TB-ACF participants on:<br><br>3.2 Numbers of newly diagnosed HIV-positive participants, and numbers subsequently linking into HIV care<br><br>3.3 Numbers of participants diagnosed with TB using sputum microscopy	To compare **between randomisation arms**:<br><br>3.1 The rate (per 1000 adult cluster residents p.a.) of investigation for TB, defined by entry into the "Presumptive TB Register" in selected routine TB Registration Facilities<br><br>**Within clusters randomised to the ACF-arm only**, to document rates (per 1000 adult cluster residents p.a.) of:<br><br>3.2 New diagnosis of HIV made by the ACF team using OraQuick, with follow-up for confirmatory testing and ART initiation<br><br>3.3 New diagnosis of TB made by the ACF team using microscopy
4. Economic and mathematical modelling analyses	4.1 to determine the costs<br>• per adult screened with microscopy during ACF<br>• per smear+ve TB patient detected by the ACF team<br>• per additional adult investigated for TB by the routine health system<br>• per additional adult diagnosed with TB by the routine health system<br>• likely final scale-up programme costs	4.1 Costing of ACF from the provider-perspective. Denominators will use records from the ACF team, and routine health system data collected by trial arm under the extended TB surveillance system.<br><br>4.2 Costing of HIV self-testing for HIV-positive clients to the TB-ACF, with denominators obtained from ACF team and facility records

	4.2 to determine the incremental costs of adding HIV to TB ACF<br>• per adult screened for HIV by the ACF team<br>• per adult with HIV detected by the ACF team<br>• per adult started on ART<br><br>4.3 to estimate the cost-effectiveness per new TB infection, TB case, and TB death averted by TB and TB+HIV ACF	4.3 Fully costed mathematical modelling of the TB-ACF intervention with and without additional HIV testing components.
5. Transmission modelling analyses	5.1 To determine the fraction of Blantyre TB transmission attributable to health facility and HIV care clinic exposure<br>5.2 To investigate the potential epidemiological impact of spatially-targeted TB control interventions and improve infection control and prevention at health facilities and HIV care clinics	5.1 Transmission modelling of genomic and TB epidemiological data

9.2. Additional pre-planned analyses

Additional pre-planned analyses are summarised in the above Table, and will include stratified sub-group analyses by gender, intended to provide better understanding of: -

- the underlying epidemiology of undiagnosed TB and HIV in Blantyre, and how this is affected by ACF as well as by secular trends due to TB and HIV Programme activities and the natural history of the HIV epidemic in Malawi
- the mechanism by which ACF may leads to change in health seeking behaviour
- the costs, cost effectiveness, and projected impact of ACF for TB and HIV on underlying TB epidemiology
- the fraction of TB transmission attributable to health centre and HIV care clinic exposure, and the potential epidemiological benefits of targeted control interventions.

9.3. Case Definitions

Outcomes will be based on the following case-definitions:

9.3.1. Undiagnosed infectious (prevalent) TB

Definite: A participant investigated because of symptoms or an abnormal chest radiograph identified during the pre-intervention survey, **and** at least **2 sputum samples testing positive** for TB (microscopy, Xpert or culture). Sputum specimens taken during follow-up can contribute to meeting this case-definition provided that at least one prevalence survey specimen was positive.

Probable: A participant investigated because of symptoms or an abnormal chest radiograph identified during the pre-intervention survey, **and 1 sputum sample testing positive** for TB (microscopy, Xpert or culture), **and either** a compatible clinical illness with decision to treat for TB on clinician's review **or** loss to clinical follow-up due to illness or death. Sputum specimens taken during follow-up can contribute to meeting this case-definition if the chest radiography is considered on review to be consistent with TB.

Uncertain TB status: A participant investigated because of symptoms or an abnormal chest radiograph identified during the pre-intervention survey, **and 1 sputum sample testing positive** for TB (microscopy, Xpert or culture) **and either** no evidence of compatible clinical illness on review **or** loss to clinical follow-up due to reasons other than illness or death.

TB excluded: A participant with **either** no indications for sputum testing, **or** investigated because of symptoms/abnormal chest radiograph but with decision not to treat for TB due to no evidence of compatible clinical illness on review and complete follow-up.

9.3.2. Other epidemiological definitions for prevalent TB

Undiagnosed: patients meeting the above case-definitions who are not already on treatment

Smear-positive: at least one sputum microscopy result, agreed on by 2 independent readers as being positive in a participant who meets one of the above case-definitions for prevalent TB

9.3.3. Routinely diagnosed TB patients

TB patients registering through the routine system do not have a standardised set of investigations for diagnosis. Although most patients have a single sputum specimen taken for the purposes of bacteriological confirmation at the time of treatment, results may be compromised by poor sputum quality, and by the known limitations of sensitivity of a single specimen.

For the purposes of the trial, registering patients will be considered **sputum-positive** if they have at least one positive sputum microscopy or Xpert test recorded, regardless of source (routine, or research laboratory), and **sputum-negative culture-positive** if *M. tuberculosis* is grown from their TB registration specimen with no other positive sputum results recorded.

Patients will be considered to have **"microbiologically-confirmed TB"** if they meet definitions for either **sputum-positive** or **sputum-negative culture-positive TB.**

10. INTERVENTIONS, METHODS AND PROCEDURES

Sensitization and mobilization will be conducted before commencement of study implementation. Blantyre District Health Office and Blantyre District Assembly stakeholders will be briefed on the study to ensure support from these key stakeholders.

Thereafter, sensitization meetings will be carried out in the catchment area of the study. This community sensitization process will be coordinated by HSAs with support from SCALE staff members. The community sensitization meetings will be aimed at:

- Identifying 6 to 10 suitable volunteers that will be supporting study activities in each intervention cluster.
- Underscoring benefits to self, family, and friends of early diagnosis of infectious TB and HIV thereby encouraging participation in the study.

10.1. Recruitment

All recruitment will be population-based, as described below, except for interview of facility attendees being investigated for TB for the qualitative and economic substudies.

10.2. Screening and Eligibility Assessment

Potential participants in the different stages of the study will be screened for eligibility according to the above criteria. Screening and recruitment logs will be kept of all potentially eligible participants, with reasons for non-participation if relevant.

10.3. Informed Consent

Written informed consent will be taken from eligible participants before any trial-specific procedures are undertaken, with witnessed thumbprint used for patients unable to read and/or write. A copy of the signed informed consent will be given to the participant, with one signed copy retained for study documentation. All patient information sheets and consent forms will be translated in to local languages. It will be clearly stated that the participant is free to withdraw from the trial at any time for any reason without prejudice to future care, and with no obligation to give the reason for withdrawal.

10.4. Procedures

10.4.1. Pre-intervention survey participants

Adult ($\geq$18 years) residents from both intervention and non-intervention clusters will be selected using random sampling from all households within the cluster boundaries, aiming for 215 participants per cluster (15,500 in total).

Following written (or witnessed if illiterate) informed consent (Appendix 1.11a&b flyer, Appendix 1.12a&b information sheet and Appendix 1.1a&b consent form), all adults in selected households will be asked to complete a standardised questionnaire including demographics, socioeconomic status, and TB symptoms (Appendix 1.5a&b household questionnaire and Appendix 1.6a&b individual questionnaire).

Mid-upper arm circumference will be measured, and household-level variables including number of adult and child members, approximate dimensions of rooms used for living and sleeping, and cooking arrangements will be recorded.

Particulate matter in the air and the concentration of carbon monoxide will be measured inside study participant's households and in the open area, using hand-held particulate matter and carbon monoxide monitors.

A random selection of participants will be asked to complete an extended questionnaire (see Appendix 1.6a&b individual questionnaire), including questions to ascertain TB knowledge, attitudes and perceptions, gender equitable norms, health service utilization and social contacts mixing.

After completing the questionnaire all participants will be given a referral card for TB screening (Appendix 1.13).

TB screening will then be offered using digital chest radiograph read immediately by an experienced radiologist trained in TB prevalence surveys, with their interpretation supported by computer assisted diagnostics (CAD) (Appendix 1.2a&b consent and Appendix 1.10 CRF). HIV testing will be offered using OraQuick HIV 1/2 and parallel finger-prick Alere (Abbott) Determine™ HIV-1/2. Positive HIV test results will be confirmed using Trinity Biotech Uni-Gold™ (Appendix 1.3a&b consent and Appendix 1.7 CRF).

Participants reporting cough, or with chest radiograph abnormality, will have two sputum samples taken for Xpert, fluorescent microscopy and MGIT culture (Appendix 1.4a&b consent and Appendix 1.8 CRF) and will be followed up with Xpert/microscopy results within 2 days, and culture results within 6 weeks (Appendix 1.9 Lab results CRF). Patients with positive TB results will have confirmatory specimens taken, while being assisted to register for TB treatment at the nearest TB Registration Centre (Appendix 1.14 referral card). A study clinic appointment with clinician's review for further management will be offered to all participants with unexplained chest radiograph abnormalities.

All participants with newly diagnosed HIV will be provided with post-test counselling and assisted to register for ART at their nearest primary care clinic (Appendix 1.15 referral card).

10.4.2. ACF intervention participants

Clusters will be visited for one to two weeks on 1 occasion by a mobile outreach team.

As previously described (21,24) ACF team members will carry out brief household visits, with door-to-door enquiry for chronic cough and leafleting (Appendix 2.1a&b information sheet). As detailed previously, all households with a symptomatic adult (≥18 years) member (including those with symptoms reported by another household member in their absence) will be provided with 2 sputum collection containers for each symptomatic individual, together with information leaflets and informed consent forms, and pictorial instructions on how to produce sputum sample (Appendix 2.1a&b information sheet and consent form and Appendix 2.4 CRF).

The ACF team will visit the household on the following day, in order to collect consent forms for cases of reported cough and specimens, which will be tested using sputum microscopy (Appendix 2.5 CRF).

An oral HIV self-testing kit will also be offered with pre-test information to each ACF participant unless already known to be HIV-positive. Kits will be distributed, and oral consent documented by thumbprint, either at the time of initial identification of chronic cough (if present in person), or through a second visit at a likely more convenient time for individuals who are not present at the time of the initial door-to-door visit. Oral consent will be obtained for HIV self-testing, documented by a thumbprint (Appendix 2.2 a&b oral consent and Appendix 2.3 a&b thumbprint consent form). Names will not be recorded and witnesses will not be used to maximise participation and ensure complete confidentiality in line with the Malawi HIV Prevention and Management Act.

Information on how to use the self-testing kit, and what steps to take will be provided in person, and post-test counselling, confirmatory testing and referral for ART if self-test positive will be managed by the ACF team (Appendix 2.8a&b self-referral form, Appendix 2.9 referral to ACF team for confirmatory testing and Appendix 2.7 referral form).

Newly diagnosed ACF participants who are HIV-positive will be asked to provide a urine sample for point-of-care LF-LAM testing for TB, with results provided immediately.

Results of sputum microscopy will be delivered back to participants at a pre-defined results collection point 2 to 4 working days after collection. Participants with positive smears who do not attend for results collection will be visited at home.

All participants with positive TB results will be assisted or accompanied to attend the nearest TB registration centre for treatment registration (Appendix 2.5 TB referral form).

10.4.3. Qualitative and economic sub-studies

Qualitative sub-studies will recruit participants being investigated for TB having reported prolonged cough to the TB ACF outreach team or primary care facilities.

Approximately 60 serial in-depth interviews will be carried out with purposively selected participants attending routine TB testing services (Appendix 4.1a&b Consent forms, Appendix 4.6a&b participant information sheets and Appendix 4.8 topic guide). Interviews will explore motivations for health-seeking, and the social and economic consequences of TB investigations and diagnosis.

In addition, approximately 20 health worker will be interviewed to probe their understanding of TB and TB testing practices (Appendix 4.2a&b Consent forms, Appendix 4.7a&b participant information sheets and Appendix 4.9 topic guide).

10.5. Enhanced surveillance of TB in Blantyre District

10.5.1. Electronic TB database

A tablet-based Electronic TB database, established in 2011 and maintained jointly by Blantyre District Health Office and MLW will be continued (and expanded to include Chiradzulu District Hospital) in order to allow subdistrict TB notification rates to be monitored. All registering patients are interviewed by the District TB Officers, and have a sputum taken for TB microscopy and culture, place of residence identified through a satellite map application, and a questionnaire including all data points required by the National TB Programme reporting system, including HIV status.

Electronic records do not include patient names: patients are managed using paper-based logs that act as the source-documents for the electronic database.

10.6. Laboratory methods

10.6.1. ACF sputum specimens

Smears will be made directly from sputum, dried, stained with Auramine-O and examined under fluorescence microscopy in a quality-assured laboratory. All positive slides and one in ten negative slides will be reread by a second reader in the central MLW/COM laboratory. Positive slides will then be confirmed with ZN staining and graded for positivity according to WHO guidelines.

10.6.2. TB sputum culture

Confirmatory sputum specimens from ACF participants found to be smear positive, all routinely registering TB patients in urban Blantyre, and Prevalence Survey participants qualifying for bacteriological investigation will be processed for culture in Mycobacterial Growth Indicator Tubes, with the residual stored at -20°C in case of contamination.

In the case of contaminated cultures, the stored specimen will be retrieved and recultured after repeat decontamination. Species identification will use MBP 64 lateral flow assays, microscopic cording, and if either of these two are negative, colony morphology, temperature and inhibition by PNB. These tests will be performed in the MLW/COM and laboratory.

Standard Operating Procedures are maintained by the MLW/COM laboratory

10.6.3. Storage of specimens

M. tuberculosis isolates will be stored for genotyping. No other specimen storage is anticipated.

DNA extraction from culture positive *Mtb* isolates will be performed in the MLW/COM laboratory by trained technicians. DNA isolates will be shipped to TGen, Arizona, USA under a material transfer agreement, where they will undergo whole genome sequencing for genomic epidemiology and TB transmission sub-studies (detailed above).

10.6.4. Laboratory Safety

Staff training will be given to all employees given regarding universal precautions and avoidance of needle-stick injuries. A policy for needle-stick injuries is in place.

The study Laboratory is a restricted access Category 2 and 3 facility with a quality management system in place, with regular external audit of laboratory procedures.

Sputum specimens handled in the field are unlikely to pose a major hazard, due to natural ventilation and the low concentration of mycobacteria in unprocessed specimens. Nonetheless, safety training will be given to all employees and universal precautions will be used to minimise the hazard of acquiring TB or other respiratory pathogens.

10.7. Discontinuation/Withdrawal of Participants

Each participant has the right to withdraw from the trial at any time. In addition, the Investigator may discontinue a participant from the trial at any time if the Investigator considers it necessary for any reason including ineligibility (either arising during the trial or

retrospectively having been overlooked at screening), significant protocol deviation or withdrawal of consent. The reason for withdrawal will be recorded in the CRF.

10.8. Definition of End of Trial

The trial will be considered closed following the completion of follow up of the last participant, and once all follow-up and laboratory reports have been received. The trial may be terminated early by the TSC, regulatory authorities or the funders, for example if interim analysis shows an unacceptably high mortality in the intervention arm, as defined in the analysis plan.

11. DATA COLLECTION AND MANAGEMENT

11.1. Data management

Data will be managed at the project offices in MLW. Data analysis will be carried out in-house in collaboration with LSHTM statisticians.

Data collection and entry will be as detailed in the trial SOPs. All data will be entered into Databases, using a combination of Electronic Data Capture onto tablets, and Optical Character Recognition (OCR) of paper forms, with validity checks before scanning, and a protocol for verification of scanned records.

Inconsistencies will be investigated and resolved. ACF and routine health system data will be analysed on completion of each respective stage of the study and for each round of intervention, with the aim being to have all data verified, double entered, checked and resolved according to the data analysis plan. The final analyses will then use the combined study data.

Access to the final data set will be limited to the Trial Coordinator, Statistician, Data Manager, and Principal Investigator.

11.2. Data capture forms and questionnaires

A full set of case-report forms (CRF)s is provided in the Appendices

11.3. Patient confidentiality

The trial staff will ensure that participants anonymity is maintained.

Participants will be identified only by a unique study identifier on the data capture tools and any electronic database. All documents will be stored securely and only accessible by trial staff and authorised personnel. The trial will comply with the Data Protection Act, which requires data to be anonymised as soon as it is practical to do so.

11.4. Data security

Hard copies of data and study documentation will be kept in locked offices, and long-term storage will be in locked cupboards in a locked repository.

Electronic copies of data will be saved in password-protected files. All data will be backed up daily by the MLW Data Office onto a physical server, and then via internet to a cloud-based server.

11.5. Quality assurance

Data will be checked for internal inconsistencies during verification and following data entry. Quality assurance protocols are in place for each stage of the study, as detailed in the individual SOPs.

For the Electronic TB Database, records will be periodically checked for completeness against Paper TB Registers, and by random selection of patients for home visit to provide ongoing QA/QC of the routinely collected TB data.

12. ADVERSE EVENT MONITORING AND REPORTING

The intervention has minimal potential for harm, with all components already conforming to International Best Practice: rather the research component relates to the impact evaluation through prevalence survey and skin testing of a public health intervention.

- Both systematic screening for TB and HIV self-testing are recommended by the WHO as International Best Practice for high HIV and TB prevalence settings, with urban Blantyre meeting both TB and HIV epidemiological criteria for community-based interventions
- Malawi National TB programme policy specifically endorses ACF in urban settings. Because of the limited sensitivity of all available TB screening tools in PLHIV, there is no single recommended screening algorithm, and the one proposed here (symptoms-then-microscopy) is one of the internationally recognised options for community-based ACF.
- Malawi National HIV Policy specifically endorses HIV self-testing in communities with high prevalence of undiagnosed HIV
- Malawi National HIV Policy specifically recommends urine LF-LAM for diagnosis of TB in symptomatic PLHIV, including outpatients

The main trial outcomes will not be available until the results of the post-intervention prevalence survey is completed, and so there is no basis from which to recommend early termination of the intervention.

As such, **we request exception from a formal Data Safety and Monitoring Board (DSMB)**, as being not indicated. The trial will instead be managed by periodic reports of progress and reporting all identified anticipated harms to a Trial Steering Committee (TSC), the Ethics Committees, and the MLW Community Advisory Group.

12.1. Anticipated harms from the intervention

Although minimal, risks from TB screening include potential for false positive and false negative HIV and TB results, and (41). These will be minimized by meticulous QA of microscopy results (blinded double reading of positives and a selection of negative smears), and by providing confirmatory testing of all provisionally smear-positive or LF-LAM positive participants.

Oral HIV self-testing is highly accurate when supported by brief in person demonstration and has a well-established strong safety record reported from Malawi (8, 36,37). No suicides have been reported from over a million self-tests in Southern Africa. The ACF team members will be trained to provide HIV self-testing in a safe, accurate and supportive way, using methods that are well established within MLW and Malawi. This will include basic psychological support and how to facilitate treatment of positive TB or HIV results (36).

12.2. Institutional responsibilities

Adverse events will be reported immediately to the Principal Investigator and will be logged and reported through regular follow-up reports.

A 6-monthly progress report will report on safety as well as other important process indicators and will be sent to the Trial Steering Committee Members, Ethics Committees, and collaborators in Ministry of Health of Malawi and Blantyre District Health Office.

12.3. Reporting Procedures

Adverse event forms will be completed by the Trial Coordinator and reported to the Principal Investigator. The Principal Investigator will check the form, make changes as necessary, sign and email a scanned copy of the form to the Ethics Committees and Sponsor.

In all cases participants experiencing adverse events will be followed up until final clinical outcome has been established (complete clinical recovery, or clinical condition has stabilised).

Adverse events will be evaluated for seriousness, and likely causality by the Principal Investigator for final classification.

The following adverse events will be systematically recorded and reported, while recognising that complete capture cannot be guaranteed during this type of public-health intervention:

- Misclassification or misinterpretation of results reported in error to a participant or their medical team, and with potential to result in TB or HIV treatment being started in error
- Serious adverse events relating to HIV self-testing, as previously defined by MLW and WHO (37)
- Withdrawal from further investigations by a participant with untreated positive sputum microbiology

12.4. Good Clinical Practice (GCP)

The principles of GCP will be adhered to throughout, and all research staff will have certified GCP training and will adhere to ICH-GCP requirements for source documentation and storage.

For the routinely-captured TB programme data, documentation to ICH-GCP standards will not be possible, and nor will written informed consent for data capture. Instead, Routine District Health Office TB Officers will be provided with protocol and modified GCP training. An established programmatic quality assurance/quality control (QA/QC) SOP will be used to maintain and understand data quality, with a verbal consent process for use of data and possible home visit for QA/QC purposes built into the Electronic TB Register.

12.5. Monitoring and audit

Project managers and supervisors will be responsible for day-to-day monitoring. Project meetings attended by all field staff will be held each fortnight, during which any problems encountered problems will be discussed and refresher training will be provided.

Internal audits will be conducted within 3 months of starting the intervention, and not less than annually during the intervention stage. These will include audit of study documentation, data storage, protocol adherence and SAE and incident reporting. A report will be circulated among TSC members and project managers. Prof Corbett will responsible for ensuring that problem areas identified during the audits are rectified, and for circulating details of actions taken to the TSC.

The LSHTM, College of Medicine Research Support Centre and MLW Research Governance Officer may carry out external audit of this study, but otherwise, no external audit will be carried out.

13. ECONOMIC EVALUATION

The main objective of the economic evaluation is to estimate the affordability and cost-effectiveness of the TB/HIV ACF intervention with the main focus being the TB intervention.

Specifically, we will estimate the incremental cost-effectiveness of ACF in comparison to facility-based case-finding only. To achieve this, inputs (costs) and outputs will be estimated.

Effectiveness data will be provided by:

1. Numbers of additional patients started on TB treatment, both a) following detection of undiagnosed patients by the ACF and b) as evidenced by differences between trial arms in notification rates identified through the Blantyre District Electronic TB Register ("additionality")
2. Numbers of **new TB cases** and **TB deaths** averted as estimated from fitting an established HIV and TB model (13) to pre-intervention and post-intervention survey data and case-notifications.

Key outputs will be:

1. Incremental costs per additional individual started on TB treatment (using both direct diagnosis by the ACF team and additionality capture by the Electronic TB Register)
2. Incremental costs per DALY, new TB case, new TB infection and TB death averted

Two economic evaluations will be undertaken: firstly, a within trial evaluation; and secondly, projection a fully-costed dynamic transmission model. Both will be used to estimate the expected incremental cost per disability-adjusted life year (DALY). The mathematical model will estimate TB infection, case and TB death averted. For each analyses, the perspective will be that of the provider, and will only include the direct intervention and medical costs.

The within trial evaluation will adopt a time horizon matching the length of intervention in the trial, with no need to discount costs or DALYs given that the intervention is brief. The model-based evaluation will adopt a 20 year horizon, with future costs and DALYs discounted at 3.5%, and sensitivity analysis to explore alternative discount rates.

The primary analysis will focus on the direct intervention and the broader healthcare costs. This will include the costs of TB and HIV screening, the costs incurred in linking participants to TB/HIV services and any associated staff costs.

The healthcare resources used will be captured prospectively and primary costing studies will be undertaken to estimate the unit costs for these identified resources. The broader healthcare resource use will be captured through interview of patients who are SCALE cluster residents (both intervention and non-intervention arms) starting TB treatment at routine facilities.

The provider-costs of treating TB will be estimated from previously collected inpatient data (43,44) and from interview of TB outpatients and District Health staff to establish:

- Outpatient clinic visits
- Days of inpatient hospital care
- Medications
- Investigations and procedures

As Malawi Ministry of Health does not have reference costs for these healthcare resources, previous unit costs estimated in Malawi will be used to derive total costs.

Mean costs and outcomes for the ACF model used in SCALE will be estimated, together with the mean incremental cost-effectiveness ratio. Measures of uncertainty (standard errors and confidence intervals) will also be reported for the mean estimates.

Sensitivity and scenario analysis will also be considered. This will include undertaking a cost-effectiveness analysis where the primary outcome will reflect two outcomes being evaluated in the trial: the proportion of participants with undiagnosed or untreated TB. As missing data is a common occurrence in trials, additional analysis will explore impact of missing data and alternative approaches to account for missing data.

14. STATISTICS

14.1. Sample size justification

Sample sizes were initially calculated assuming a decline from the last National TB Prevalence Survey in 2013-14 given that there has been case-finding activity since then. However, the interim results from a **pre-intervention survey** show greater than anticipated decline (target of 14,511 participants will give precision of +/- 2.5 around an assumed true prevalence of 900 per 100,000 adults, assuming design effect of 1.5 from random household sampling. The increase to 15,500 allows for wastage from e.g. unreadable Xray/contaminated cultures).

Sample size calculations and statistical analyses will use established approaches for cluster-randomised trials.

The **primary trial outcome** has been changed from self-reported testing outcome to TB case notification.

For this, we assume an annual case-notification of adult microbiologically-confirmed TB to be in the range of 70 to 100 per 100,000 adults for the standard of care clusters, based on current data and trends.

An adult population of ~150,000 per arm followed over 12 months will then provide power to detect a 44% to 56% increase in microbiologically confirmed TB if $k = 0.3$ to 0.4 .

Analysis will use standard methods appropriate for cluster-randomised trials, with adjustment for pre-intervention rates of adult microbiologically-confirmed TB.

14.2. Data analysis plan

A detailed statistical analysis plan will be written and approved by the TSC prior to completion of enrolment.

15. STUDY ADMINISTRATION AND INSTITUTIONAL RESPONSIBILITIES

The trial will be conducted in accordance with the current approved protocol. The Investigator will ensure that this trial is conducted in accordance with the principles of the Declaration of Helsinki, with the ICH Guidelines for Good Clinical Practice (CPMP/ICH/135/95) July 1996, UK Medical Research Council (MRC) Guidelines for GCP and relevant regulations and standard operating procedures (SOPs).

All study data collected will be subject to random sampling for verification and accuracy in relation to source documents, in addition to data quality checks inbuilt to the data recording systems. Any problems with data quality will be reported to the TSC and appropriate action taken, including increasing frequency of checks.

Laboratory testing will undergo periodic quality assurance procedures including proficiency testing of study diagnostics. All data monitoring and quality assurance processes will be outlined in detail in a separate SOP and will be compliant with ICH GCP and MRC guidance.

Regular monitoring will be performed according to ICH GCP.

15.1. Funding

The trial is funded by the Wellcome Trust.

15.2. Trial Sponsor and insurance

The London School of Hygiene and Tropical Medicine (LSHTM) will act as the main sponsor for the study and holds public liability and clinical trial insurance policies which apply to this study, which would operate in the event of any participant suffering harm as a result of their involvement in the research.

15.3. Trial Steering Committee

The trial steering committee (TSC) will oversee the trial, monitor progress, advise the PI and investigator team, and report to the trial funders and sponsor. The TSC will include and independent Chairman and 2 independent members as well as the investigators.

The TSC will meet prior to the trial start and 6-monthly thereafter to monitor progress.

15.4. Data Safety and Monitoring Board

For the reasons outlined in the Chapter on Safety Monitoring and Reporting section, above, we request exception from a formal Data Safety and Monitoring Board (DSMB) due to the public-health nature of the intervention, which is already full endorsed by national and international policy, and because the only data from which to judge effectiveness are from the post-intervention survey.

16. ETHICAL CONSIDERATIONS

16.1. Ethical Approvals

The protocol, informed consent form, participant information sheet and any proposed advertising material will be submitted to Research Ethics Committees (REC) of the London School of Hygiene & Tropical Medicine, and the College Of Medicine Research and Ethics Committee (COMREC).

The Principal Investigator will submit and, where necessary, obtain approval from the above parties for all substantial amendments to the original approved documents.

There are a number of potential benefits for participants SCALE, including the potential to receive early diagnosis and treatment for HIV and TB. Nevertheless, we have carefully considered potential harms for participants, and will inform participants about potential risks.

16.2. Informed consent at the different stages of the study

As detailed in the Methods section, written or witnessed informed consent will be taken from all participants included in the 2 surveys that are part of the evaluation of the intervention trial as well as the sputum TB testing component of the ACF.

For the HIV self-testing component of the ACF, we request an adaptation of informed consent procedures: using verbal consent documented by unnamed thumbprint. This is because:

- There is minimal potential for harm from mouth swab HIV self-testing
- HIV self-testing is endorsed by the Malawi Ministry of Health with no requirement for written consent
- HIV self-testing is now part of international (WHO) policy, also with no requirement for written consent
- This fully complies with the recent Malawi National HIV Prevention and Management Act.

We also request **verbal informed consent** for collection of electronic data and a sputum specimen from all routine registering TB patients and patients being investigated for TB, as these activities are routine activities of TB Officers who are Ministry of Health employees

16.3. Participant compensation

Due to the high number of survey participants (planned sample size of 83,900) participant compensation will be limited to **MKw 1000 per participant.** No travel is needed for all but the small number of participants who require clinical follow-up for confirmed TB or newly diagnosed HIV infection, or respiratory illness that is TB-test negative. Assistance with transport money for those needing clinic visit will be provided.

16.4. Risks and risk-minimisation

With any screening test, there is potential for false-positive and false-negative results. These risks will be minimized by confirming all positives before treatment of HIV and TB, and by warning participants that a negative TB test does not fully exclude TB.

For the prevalence survey, radiological screening (adults) will be used which has minimal risks.

A single **chest x-ray** typically delivers an average effective dose of 0.01mSv, comparable to 10 days of natural background radiation, and with less than one in a million chance of causing cancer. The potential benefits of chest x-ray as a TB diagnostic are then likely to

outweigh this very small risk and are safe in pregnancy. Abdominal shielding will be used for women of child-bearing age.

Confidentially will be maintained for all survey participants, with this need emphasized in all protocol and GCP trainings. All HIV testing and TB screening activities will be undertaken in private and out of hearing of others, using tents and screens.

All adults newly identified by the study as having TB disease, or HIV infection are in need of immediate treatment under Malawi National Guidelines and will be supported to register and access routine treatment services.

16.5. Capacity-building

The 3 year trial will support one LSHTM PhD Fellowship to a Malawian Research Epidemiologist (McEwen Khundi) and one distance-based MSc in Epidemiology from LSHTM (Rebecca Nzawa). Further capacity development in DNA extraction will be provided to MLW/COM TB lab technicians to ensure TB whole genome sequencing can be conducted in-country for future studies.

17. DISSEMINATION

Regular meetings will be held with TB Officers, Blantyre DHO staff, and the National TB Programme.

MLW has an active Community Advisory Group that meets regularly, and a Science Communication Programme that will be able to support Science Cafes and that holds regular radio programmes and other community engagement activities.

17.1. Public engagement and scientific dissemination

We will share study results with participants, community members and facility health workers at a public dissemination to be held either at MLW or at a community venue following completion of all study activities. This will allow key stakeholders to gain first insight into the study results, and to ask questions about how the study findings will be further disseminated and acted upon.

Trial results will be shared with the Blantyre District Health Office, Malawi National Tuberculosis Programme and with the Malawi National HIV Programme.

We will report findings at national, regional and international conferences, and will submit a manuscript reporting trial findings to peer-reviewed journals.

17.2. Translating Research into Policy

Results of this research will be important in guiding national, regional and international health policy as international and national policymakers and parliamentarians works towards meeting agreed 2022-2035 EndTB Targets in Malawi and elsewhere in the region.

A key objective of SCALE is to provide research findings to support the wider implementation of affordable and effective interventions.

The Principal Investigator of SCALE has already had substantial influence on International Policy and Practice for TB screening and ACF through the 2013 WHO Guidelines, and on National TB Programme Policy in Malawi through the 2016 Malawi National TB Research Network. She is well connected to the Malawi Ministry via both HIV and TB Technical Working Groups. The National TB Programme Manager is a co-investigator on this proposal.

18. STUDY TIMELINE

The SCALE CRT is funded for 3 years, from Sept 2018 to August 2021.

19. STUDY BUDGET

The total grant funding is £4.15m with £1.99m of that allocated for in-country resources in Malawi.

Staff in Malawi include the PI, Trial Coordinator, Research Epidemiologist, Field Trial Coordinator, Community Liaison Officer, Data manager, assistant Data Manager and 2 Data Clerks, Spatial data officer, Senior field officer, Social scientist FTE for 2 years, Administrator, Financial Clerk and 3 drivers, TB Laboratory staff include a Laboratory Manager, 3 technicians, 4 microscopists and a lab cleaner. Additional team members will be employed for the post intervention survey (6 nurses for tuberculin survey, 3 Radiographers, as well as temporary enumerators, data staff, and community health workers employed on a MoH scale. The ACF intervention will be delivered by 8 community workers.

20. REFERENCES

1. World Health Organization. Global Tuberculosis Report 2018. Geneva 2018.
2. Suthar AB, Lawn SD, del Amo J, Getahun H, Dye C, Sculier D, et al. Antiretroviral therapy for prevention of tuberculosis in adults with HIV: a systematic review and meta-analysis. *PLoS Med*. 2012;9:e1001270.
3. Williams BG, Granich R, De Cock KM, Glaziou P, Sharma A, Dye C. Antiretroviral therapy for tuberculosis control in 9 African countries. *Proc Natl Acad Sci USA*. 2010;107:19485-9.
4. Getahun H, Kittikraisak W, Heilig CM, Corbett EL, Ayles H, Cain KP, et al. Development of a standardized screening rule for tuberculosis in people living with HIV in resource-constrained settings: individual participant data meta-analysis of observational studies. *PLoS Med*. 2011;8:e1000391.
5. World Health Organization. Systematic screening for active tuberculosis: principles and recommendations. Geneva 2013.
6. Chikovore J, Hart G, Kumwenda M, Chipungu GA, Desmond N, Corbett EL. Control, struggle, and emergent masculinities: a qualitative study of men's care-seeking determinants for chronic cough and tuberculosis symptoms in Blantyre, Malawi. *BMC Public Health*. 2014;14:1053
7. Druyts E, Dybul M, Kanters S, Nachega J, Birungi J, Ford N, Thorlund K, Negin J, Lester R, Yaya S, Mills EJ. Male sex and the risk of mortality among individuals enrolled in antiretroviral therapy programs in Africa: a systematic review and meta-analysis. *AIDS*. 2013; 28;27:417-25.
8. Choko AT, MacPherson P, Webb EL, Ball H, Sambakunsi R, Mdolo A, Makombe SD, Desmond N, Hayes R, Maheswaran H, Corbett EL. Uptake, accuracy, safety and linkage into care over two years of promoting annual self-testing for HIV in Blantyre, Malawi: a community-based prospective study. *PLoS Med* 2015;12:e1001873.
9. Corbett EL, MacPherson P. Tuberculosis screening in high HIV prevalence settings: turning promise into reality. *Int J Tuberc Lung Dis*. 2013 Sep;17(9):1125-38.
10. Knight GM, Dodd PJ, Grant AD, Fielding KL, Churchyard GJ, White RG. Tuberculosis prevention in South Africa. *PLoS One*. 2015;10:e0122514.
11. Vynnycky E, Sumner T, Fielding KL, Lewis JJ, Cox A, Hayes RJ, Corbett EL, Churchyard GJ, Grant AD, White RG. Tuberculosis control on South African goldmines: mathematical modelling of a trial of community-wide isoniazid preventive therapy. *Am J Epi* 2015 15;181:619-32.
12. Kanyerere H,Mganga A, Harries AD, Tayler-Smith K, Jahn A, Chimbwandira FM, Mpunga J. Decline in national tuberculosis notifications with national scale-up of antiretroviral therapy in Malawi. *Public Health Action* 2014; 4: 113–115
13. Dodd PJ, Knight GM, Corbett EL, Lawn SD, White RG. Predicting the long-term impact of ART scale-up on population incidence of tuberculosis. *PLoS One* 2013. 8(9):e7546.
14. Godfrey-Faussett P, Sonnenberg P, Shearer SC, Bruce MC, Mee C, Morris L, Murray J. Tuberculosis control and molecular epidemiology in a South African gold-mining community. Lancet. 2000;356:1066-71.
15. Dodd PJ, Looker C, Plumb ID, Bond V, Schaap A, Shanaube K, Muyoyeta M, Vynnycky E, Godfrey-Faussett P, Corbett EL, Beyers N, Ayles H, White RG. Age- and Sex-Specific Social Contact Patterns and Incidence of Mycobacterium tuberculosis Infection. *Am J Epidemiol* (2016) 183(2):156-66
16. Horton K, MacPherson P, Houben R, White R, Corbett EL. Sex Differences in Tuberculosis Burden and Notifications in Low- and Middle-Income Countries: A Systematic Review and Meta-analysis. *PLoS Med* (2016) 13(9):e1002119
17. Dye C, Bassili A, Bierrenbach AL, Broekmans JF, Chadha VK, Glaziou P, et al. Measuring tuberculosis burden, trends, and impact of control programmes. *Lancet Inf Dis*. 2008;8:233-43

18. Dodd PJ, White RG, Corbett EL. Periodic active case finding for TB: when to look? *PLoS One*. 2011;6(12):e29130.
19. Golub JE, Mohan CI, Comstock GW, Chaisson RE. Active case finding of tuberculosis: historical perspective and future prospects. *Int J Tuberc Lung Dis*. 2005;9:1183-203.
20. Kranzer K, Afnan-Holmes H, Tomlin K, Golub JE, Shapiro AE, Schaap A, Corbett EL, Lönnroth K, Glynn JR. The benefits to communities and individuals of screening for active tuberculosis disease: a systematic review. *Int J Tuberc Lung Dis*. 2013 Apr;17(4):432-46.
21. Corbett EL, Bandason T, Duong T, Dauya E, Makamure B, Churchyard GJ, et al. Comparison of two active case-finding strategies for community-based diagnosis of symptomatic smear-positive tuberculosis and control of infectious tuberculosis in Harare, Zimbabwe (DETECTB): a cluster-randomised trial. *Lancet*. 2010;376(9748):1244-53.
22. Victora CG, Habicht J-P, Bryce J. Evidence-based public health: moving beyond randomised trials. *Am J Pub Hlth* 2004; 94:400-5
23. Marks GB, Phuong NTB, Anh NT, Hoa NB, Khanh LB, Ho J, Fox GJ, Nhung NV. The effect of population-wide screening on the prevalence of tuberculosis: a cluster-randomised trial in Vietnam. 49th World Conference on Lung Health, 2018, The Hague, Netherlands.
24. Nliwasa M, Choko A, Mwapasa M, Grint D, MacPherson P, Corbett EL. Trends In HIV And Sex-Stratified Tuberculosis Case Notifications In Blantyre, Malawi (ID 1636). Poster 2018 Conference on Retroviruses and Opportunistic Infections (CROI), Boston, Massachusetts, USA.
25. Creswell J, Codlin AJ, Andre E, Micek MA, Bedru A, Carter EJ, Yadav RP, Mosneaga A, Rai B, Banu S, Brouwer M, Blok L, Sahu S, Ditiu L. Results from early programmatic implementation of Xpert MTB/RIF testing in nine countries. *BMC Infect Dis*. 2014;14:2.
26. Yassin MA, Datiko DG, Tulloch O, Markos P, Aschalew M, Shargie EB, Dangisso MH, Komatsu R, Sahu S, Blok L, Cuevas LE, Theobald S. Innovative community-based approaches doubled tuberculosis case notification and improves treatment outcome in Southern Ethiopia. *PLoS One*. 2013 May 27;8(5):e63174.
27. Ayles H, Muyoyeta M, Du Toit E, Schaap A, Floyd S, Simwinga M, Shanaube K,Chishinga N, Bond V, Dunbar R, et al. Effect of household and community interventions on the burden of tuberculosis in southern Africa: the ZAMSTAR community-randomised trial. *Lancet*. 2013;382:1183-94.
28. World Health Organization. Community-directed interventions for priority health problems in Africa: results of a multicountry study. *Bull World Health Organ*. 2010;88(7):509-18
29. van't Hoog AH, Cobelens F, Vassall A, van Kampen S, Dorman SE, Alland D, Ellner J. Optimal triage test characteristics to improve the cost-effectiveness of the Xpert MTB/RIF assay for TB diagnosis: a decision analysis.PLoS One. 2013 Dec 18;8(12):e82786. doi: 10.1371/journal.pone.0082786. eCollection 2013.
30. Monk E, Kumwenda MK, Nliwasa M, Mpunga J, Corbett EL. Factors affecting tuberculosis health message recall two years after active case-finding in Blantyre, Malawi. IJTLD 2018 22(9):1007-1015. DOI: 10.5588/ijtld.18.0006; PMID: 30092865 Open Access
31. Murray M, Cattamanchi A, Denkinger C, Van't Hoog A, Pai M, Dowdy D. Cost-effectiveness of triage testing for facility-based systematic screening of tuberculosis among Ugandan adults. BMJ Glob Health. 2016 Sep 28;1(2):e000064. doi: 10.1136/bmjgh-2016-000064. eCollection 2016.
32. World Health Organization. Chest Radiology in Tuberculosis Detection: Summary of recommendation and guidance on programmatic approaches. Geneva, 2016.

33. Pande T, Cohen C, Pai M, Ahmad Khan F. Computer-aided detection of pulmonary tuberculosis on digital chest radiographs: a systematic review. Int J TB Lung Dis 2016; 20(9): 1226-30.
34. World Health Organization. TB prevalence surveys: a handbook. Geneva 2011.
35. Nliwasa, M, MacPherson P, Gupta-Wright A, Mwapasa M, Horton K, Odland JO, Flach C, Corbett EL. High HIV and active tuberculosis prevalence and increased mortality risk in adults with symptoms of TB: a systematic review and meta-analyses. J Int AIDS Soc 2018; 21(7):e25162.
36. Hatzold K, Gudukeya S, Mutseta M, Chilongosi R, Nalubamba M, Nkhoma C, Munkombwe H, Munjoma M, Mkandawire P, Mabhunu V, Smith G, Madidi N, Ahmed H, Kambeu T, Stankard P, Johnson C, Corbett EL. HIV Self-Testing: Breaking the barriers to uptake of testing among men and young people in sub-Saharan Africa, experiences from STAR demonstration projects in Malawi, Zambia and Zimbabwe. J Int. AIDS Soc. (in press).
37. Kumwenda M, Johnson C, Choko A, Lora W, Sibande W, Sakala D, Indravudh P, Chilongosi R, Baggaley R, Nyirenda R, Taegtmeyer M, Hatzold K, Desmond N, Corbett EL. Exploring social harms and HIV self-testing using mixed-methods in Malawi. J Int. AIDS Soc. (in press)
38. Peter JG, Zijenah LS, Chanda D, et al. Effect on mortality of point-of-care, urine-based lipoarabinomannan testing to guide tuberculosis treatment initiation in HIV-positive hospital inpatients: a pragmatic, parallel-group, multicountry, open-label, randomised controlled trial. Lancet 2016; 387(10024): 1187-97.
39. Gupta-Wright A, Corbett, E.L, van Oosterhout, JJ, Wilson D, Grint D, Alufandika-Moyo M, Peters JA, Chiume L, Flach C, Lawn SD, Fielding K. Rapid urine-based screening for tuberculosis in HIV-positive patients admitted to hospital in Africa (STAMP): a pragmatic, multicentre, parallel-group, double-blind, randomised controlled trial. Lancet (2018) ; DOI: 10.1016/S0140-6736(18)31267-4;
40. Aggerbeck H, Ruhwald M, Hoff ST, Borregaard B, Hellstrom E, Malahleha M, Siebert M, Gani M, Seopela V, Diacon A, Lourens M, Andersen P, Dheda K. C-Tb skin test to diagnose *Mycobacterium tuberculosis* infection in children and HIV-infected adults: A phase 3 trial. PLoS One. 2018 Sep 24;13(9):e0204554.
41. Dowdy DW, Gounder CR, Corbett EL, Ngwira LG, Chaisson RE, Merritt MW. The ethics of testing a test: randomized trials of the health impact of diagnostic tests for infectious diseases. *Clin Infect Dis*. 2012;55:1522-6.
42. Maheswaran H, Petrou S, MacPherson P, Choko AT, Kumwenda F, Lalloo D, Clarke A, Corbett EL. The economic and quality of life impact of HIV self-testing versus facility-based HIV testing and counselling in Blantyre, Malawi. *BMC Medicine* (in press)
43. Maheswaran, H. ; Petrou, S. ; Cohen, D. ; MacPherson, P. ; Kumwenda, F. ; Lalloo, D.G. ; Corbett, E.L. ; Clarke, A. ; Economic costs and health-related quality of life outcomes of hospitalised patients with high HIV prevalence: A prospective hospital cohort study in Malawi. PLoS One (2018) 13(3):e0192991.
44. Maheswaran, H. ; Clarke, A. ; MacPherson, P. ; Kumwenda, F. ; Lalloo, D.G. ; Corbett, E.L. ; Petrou, S.; Cost-Effectiveness of Community-based Human Immunodeficiency Virus Self-Testing in Blantyre, Malawi Clin Infect Dis (2017) ; DOI: 10.1093/cid/cix983; PMID: 29136117 Open Access

APPENDIX 1 – PRE-PREVALENCE SURVEY TOOLS

Consent Forms

1.1.a. TS05FMa Consent form for TB Prevalence Survey - English
1.1.b. TS05FMb Consent form for TB Prevalence Survey - Chichewa

1.2.a. TS09a X-ray Consent Form - English
1.2.b. TS09b X-ray Consent Form – Chichewa

1.3.a. TS17a HTS Consent Form - English
1.3.b. TS17b HTS Consent Form - Chichewa

1.4.a. TS21a Sputum Collection Verbal Consent - English
1.4.b. TS21b Sputum Collection Verbal Consent – Chichewa

CRFs

1.5.a. TS06QE Pre-Prevalence Household Questionnaire – English
1.5.b. TS06QE Pre-Prevalence Household Questionnaire – Chichewa

1.6.a. TS07QE Pre-Prevalence Individual Questionnaire – English
1.6.b. TS07QE Pre-Prevalence Individual Questionnaire - Chichewa

1.7. TS24FM HTS Result Form
1.8. TS15FM Sputum Collection Form
1.9. TS15FMb TB Culture Results Form – Lab
1.10. TS23FM X-ray Form

Flyers

1.11.a. TS01a General Flyer TB Prevalence Survey – English
1.11.b. TS01b General Flyer TB Prevalence Survey – Chichewa

Participant Information Sheets

1.12.a. TS02a Pre-Intervention Prevalence Survey Participant Information Sheet –English
1.12.b. TS02a Pre-Intervention Prevalence Survey Participant Information Sheet – Chichewa

Referral Cards

1.13. TS13FM Tent patient held referral card
1.14. TS33FM TB Facility Referral Form
1.15. TS35FM HTS Referral Form
1.16. TS36FM X-ray Clinic Referral Form

APPENDIX 2 – ACF TOOLS

Consent Forms

2.1.a. CF01FLa ACF Information Sheet and Consent Form - English
2.1.b. CF01FLb ACF Information Sheet and Consent Form – Chichewa

2.2.a. CF03a HIV Self-testing Oral Consent – English
2.2.b. CF03b HIV Self-testing Oral Consent - Chichewa

2.3.a. CF04a HIV Self-testing Thumbprint Consent Form – English
2.3.b. CF04b HIV Self-testing Thumbprint Consent Form - Chichewa

CRFS

2.4. CF09a Sputum Form1
2.5. CF09b Sputum Form2

Referral cards

2.6. CF10FM TB Facility Referral Form
2.7. CF21FM HTS Referral Form

2.8.a. CF71FMa HTS Self-Referral Form - English
2.8.b. CF71FMb HTS Self-Referral Form - Chichewa

2.9. CF72FM HTS Confirmatory Referral Form

APPENDIX 3 – HEALTH FACILITY TB TESTING TOOLS

Consent forms

3.1.a. Tu04FMa Consent form for Presumptive TB Patient Interviews –English
3.1.b. Tu04FMb Consent form for Presumptive TB Patient Interviews –Chichewa

3.2.a. Tu06FMa Consent form for HCW Interviews – English
3.2.b. Tu06FMb Consent form for HCW Interviews – Chichewa

3.3.a. TU07FMa Oral Consent for Presumptive TB Electronic register – English
3.3.b. TU07FMb Oral Consent for Presumptive TB Electronic register – Chichewa

CRFs

3.4. Tu02Qa Presumptive TB patient testing electronic register

Participant Information Sheet

3.5.a. TU01a Participant Information Sheet for presumptive TB electronic register – English
3.5.b. TU01b Participant Information Sheet for presumptive TB electronic register – Chichewa

3.6.a. TU03a Participant Information Sheet for presumptive TB patient interviews – English
3.6.b. TU03b Participant Information Sheet for presumptive TB patient interviews – Chichewa

3.7.a. TU05a Participant Information Sheet for HCW interviews-English
3.7.b. TU05b Participant Information Sheet for HCW interviews-Chichewa

Topic Guides

3.8. Topic Guide Exploring motivations for and experiences of testing among presumptive TB patients
3.9. Topic Guide Exploring motivations for and experiences of testing for TB among outpatient HCW

APPENDIX 4 – MATERIAL TRANSFER AGREEMENT
